# Non-Competitive AMPA Receptor Antagonist Perampanel Inhibits Ischemia-Induced Neurodegeneration and Behavioral Deficits in Focal Cortical Pial Vessel Disruption Stroke Model

**DOI:** 10.3390/cells14201628

**Published:** 2025-10-19

**Authors:** Michael G. Zaki, Mohamed Taha Moutaoufik, Mahboubeh Pordeli, Mohan Babu, Changiz Taghibiglou, Francisco S. Cayabyab

**Affiliations:** 1 Department of Surgery, Neuroscience Research Cluster, College of Medicine, University of Saskatchewan, 107 Wiggins Road, Saskatoon, SK S7N 5E5, Canada; mgz156@mail.usask.ca (M.G.Z.);; 2 Department of Chemistry and Biochemistry, University of Regina, Regina, SK S4S 0A2, Canada; mohamed.moutaoufik@um6p.ma (M.T.M.); mohan.babu@uregina.ca (M.B.); 3 Department of Anatomy, Physiology and Pharmacology, College of Medicine, University of Saskatchewan, 107 Wiggins Road, Saskatoon, SK S7N 5E5, Canada; changiz.taghibiglou@usask.ca

**Keywords:** glutamate excitotoxicity, ischemic stroke, AMPA receptors, neuroprotective therapy, perampanel, long-term potentiation

## Abstract

Glutamate receptors represent a potential target for neuroprotection in neurodegenerative neurological conditions. Perampanel, a non-competitive α-amino-3-hydroxy-5-methyl-4-isoxazole propionate receptor (AMPAR) antagonist, is clinically approved for the management of epilepsy. Perampanel’s neuroprotective effects have been reported in global and focal cerebral ischemia models, but the cellular mechanisms remain incompletely understood. Therefore, we studied the potential neuroprotective effects of perampanel in rats using the pial vessel disruption (PVD) stroke model, an established focal cortical non-reperfusion ischemic stroke model. Perampanel was given once intraperitoneally (3 mg/kg body weight) 1 h after PVD surgery and repeated on days 2–3 post-surgery. On the fourth day post PVD, animal behavioral assays and imaging, biochemical, and electrophysiological analyses were performed. Compared to vehicle control, perampanel in PVD-treated rats significantly inhibited hippocampal neurodegeneration and long-term potentiation deficits. Perampanel also attenuated PVD-induced motor deficits, depressive/anxiety-like behaviors, and hippocampal-dependent cognitive impairment. In addition, perampanel prevented the PVD-induced downregulation of surface-expressed GluA1 and GluA2 AMPARs and increased phosphorylation of GluA1 at S831 and S845. Molecular docking analysis revealed perampanel binding to transmembrane regions M1, M3 and M4 of GluA1 and GluA2 subunits. Together, our results show that perampanel attenuated PVD-induced neurodegeneration and behavioral deficits by blocking AMPARs and decreasing GluA1 and GluA2 internalization. In addition, this study shows the neuroprotective potential of perampanel through the inhibition of neuroinflammation mediated by activated microglia and astrocytes following cerebral ischemia. This study is the first to evaluate perampanel in the pial vessel disruption model of ischemia without reperfusion, a clinically relevant stroke paradigm that differs fundamentally from middle cerebral carotid artery occlusion and photothrombosis stroke models.

## 1. Introduction

Stroke remains a major global health challenge, representing the second leading cause of death worldwide [1,2]. In 2019, it accounted for approximately 11.6% of all deaths. Ischemic stroke, which results from a temporary or permanent interruption of blood flow to the brain, is the most common type, responsible for about 62.4% of all stroke cases [3]. This condition often leads to significant neurological damage, contributing to long-term disability and placing a substantial burden on healthcare systems and economies [4]. Although the primary therapeutic goal of ischemic stroke management is rapid restoration of the cerebral blood flow through thrombolytics, recombinant tissue plasminogen activator (rt-PA) is the only Food and Drug Administration (FDA)-approved drug for acute ischemic stroke [5]. Notably, rt-PA has limited success not only due to its narrow therapeutic window when it must be administered <4.5 h from stroke onset but also because it is not recommended for patients older than 80 years with comorbidities [6,7]. Accordingly, further investigation into the pathways involved in stroke-induced neurodegeneration could lead to the development of neuroprotective drugs as adjunctive therapy with rt-PA.

Recently, neuroprotection has been introduced as an alternative pharmacological approach for the treatment of stroke, which helps to extend the therapeutic window, thereby reducing neuronal loss and cognitive dysfunction [8]. However, most neuroprotective agents, including glutamate antagonists, antioxidants, immunosuppressants, sodium channel blockers, and calcium channel blockers, have failed in clinical trials, showing little or no significant improvement [9,10] despite demonstrating efficacy as neuroprotective agents in animal stroke models [11,12].

Glutamate is the major excitatory neurotransmitter that mediates synaptic transmission and many physiological processes, including long-term potentiation (LTP), through its action on two classes of glutamate receptors: G-protein coupled metabotropic glutamate receptors (mGluRs) and ionotropic glutamate receptors (iGluRs), including N-methyl-D-aspartate (NMDA), α-amino-3-hydroxy-5-methylisoxazole-4-propionate receptors (AMPARs), and kainate receptors (KAR) [13]. Glutamate receptors are implicated in numerous neurodegenerative neurological conditions, including stroke, Parkinson’s disease, Alzheimer’s disease, epilepsy, and anxiety [14]. However, excessive release of glutamate during ischemia or hypoxic conditions mediates a phenomenon known as glutamate excitotoxicity [15] linked to neurodegeneration in stroke [16,17] and other neurological diseases, such as Alzheimer’s disease [18] and Parkinson’s disease [19]. Ischemic stroke results in a rapid increase in extracellular glutamate in the brain reaching toxic concentrations of 100 µM–10 mM and 30–50 µM in ischemic core and ischemic penumbra, respectively [16,17,20]. Numerous studies from animal stroke models have shown neuroprotection achieved by administration of AMPAR antagonists, such as NBQX [21], YM872 [22], and talampanel (GYKI-53405) [23]; however, these compounds have failed in clinical trials due to poor water solubility, low bioavailability, or nephrotoxicity [24,25].

Ischemia also causes extracellular adenosine elevation of up to 100-fold as a result of extracellular ATP breakdown by extracellular nucleotidases and adenosine extrusion through equilibrative nucleoside transporters of ischemic cells [26,27]. Traditionally, the inhibitory adenosine A1 receptors (A1Rs) are believed to be neuroprotective by preventing excitotoxicity via inhibition of presynaptic glutamate release and reduction in postsynaptic membrane excitability [28,29,30]. In contrast, the excitatory adenosine A2A receptors (A2ARs) increase the excitotoxicity and neuronal death, as A2AR stimulation enhances glutamatergic synaptic transmission [28,31]. However, the neuroprotective effect of A1R is short-lived because of the chronic A1R stimulation and subsequent A1R desensitization accompanying the elevated extracellular adenosine [28]. More recently we demonstrated that chronic A1R stimulation by daily intraperitoneal injections of an A1R-selective agonist caused neurodegeneration in the hippocampus and substantia nigra, brain regions where A1R and A2AR are highly expressed [32]. We also previously reported that ischemic stroke, induced by focal cortical pial vessel disruption (PVD) representing a small vessel stroke model, dramatically reduced A1R surface expression but upregulated A2AR surface expression [29]. Thus, studies in our lab focused on exploring the neuroprotective targets in cerebral ischemia through neuromodulation of adenosine signaling and glutamate excitotoxicity either through antagonism of A2AR or AMPAR. Moreover, the ischemic lesion caused clathrin-mediated internalization of both GluA1 and GluA2 subunits of AMPARs through an A1R-dependent mechanism [29]. Recently, we showed that prolonged A1R stimulation during the hypoxic/reperfusion injury model caused upregulation of calcium-permeable AMPARs, synaptic potentiation, and hippocampal neuronal damage resulting from signaling interactions between A1R and A2AR [31]; thus, blocking AMPARs can be a potential target to prevent cerebral ischemia-induced neurodegeneration. More recently, we demonstrated that istradefylline, an FDA-approved selective adenosine A2AR inhibitor as an add-on treatment for Parkinson’s disease, effectively prevented hippocampal neuronal cell death, attenuated astrocyte- and microglia-mediated neuroinflammation, and ameliorated cognitive impairment and motor deficits in this PVD-induced focal cortical ischemia model in rats [33]. The present study aimed to investigate the hypothesis that antagonism of AMPA receptors would mitigate neurodegeneration after cerebral ischemia.

Apart from the crucial role of AMPARs in synaptic plasticity and synaptic transmission, the AMPA receptors of microglia and macrophages contribute to the release of pro-inflammatory cytokines upon activation [34,35]. Moreover, AMPA receptors are expressed in inflammatory/immune cells, where they have a role in proliferation, cell adhesion, chemotaxis, and release of pro-inflammatory cytokines, such as tumor necrosis factor-α (TNF-α), and nitric oxide from inducible nitric oxide synthase (iNOS) [36,37]. In the present study, we aimed to study how AMPA receptor antagonism contributes to the attenuation of pro-inflammatory factors and restoration of the levels of anti-inflammatory cytokines following cerebral ischemia.

Perampanel (Fycompa^®^), a non-competitive AMPAR antagonist, has been approved by the FDA for anti-seizure treatment [38,39,40,41]. We have recently shown that perampanel exhibited neuroprotection and prevented the adenosine-induced synaptic potentiation observed after normoxic reperfusion following a 20 min hypoxic insult [31]. Therefore, we hypothesize that the clinically approved anti-epileptic perampanel may show efficacy as a neuroprotective agent in an in vivo cerebral ischemia stroke model. Results of the current study suggest that perampanel could be repurposed as a neuroprotective therapy to attenuate the cognitive, mood, and motor dysfunction; prevent LTP deficits; and reduce neurodegeneration in our preclinical ischemic/non-reperfusion stroke model.

## 2. Materials and Methods

### 2.1. Animal Subjects

This work was approved by the University of Saskatchewan’s Animal Research Ethics Board and complied with the Canadian Council on Animal Care guidelines for humane animal use (Approved Animal Use Protocol Number: 20070090). All experimental design, analysis, and reported number of research animals used also adhered to the ARRIVE guidelines for reporting experiments involving animal use to ensure all efforts were made to minimize animal suffering and the number of animals used in this study [42]. Male Sprague-Dawley rats at 20–30 days old (Charles River Canada, Montreal, Canada) were used in all studies. The animals were acclimated for at least 1 week prior to any experimental procedures and housed two per cage in a temperature controlled (21 °C) rat suite on a 12/12 h light/dark cycle. Rats were randomly divided into three groups (N = 15 in each group): 1. the control group (Sham), 2. the PVD/vehicle control (referred to as PVD in Figures), and 3. PVD/Perampanel (referred to as PER). The experimental design, surgical procedures, and behavioral assessments used in this study were conducted as previously described in our recent publication [33] (summarized in Supplementary Figure S1).

### 2.2. Hippocampal Slice Preparation

On the fourth day after PVD surgery, male Sprague Dawley rats from 3 groups (Sham, PVD + DMSO, and PVD + Perampanel) were anesthetized with halothane and rapidly decapitated, and the excised brains were immediately transferred into an oxygenated, ice-cold high-sucrose dissection medium (87 mM NaCl, 25 mM NaHCO_3_, 25 mM glucose, 75 mM sucrose, 2.5 mM KCl, 1.25 mM NaH_2_PO_4_, 7.0 mM MgCl_2_, and 500 μM CaCl_2_ [43]). Ipsilateral and contralateral hippocampal slices (400 μm thick) were prepared using a vibratome tissue slicer (VTS1200S, Vibram Instruments, Germany) in the same ice-cold oxygenated dissection medium as above. Prior to treatments, brain slices were incubated for 1 h at room temperature in oxygenated artificial cerebrospinal fluid (aCSF) (126 mM NaCl, 2.5 mM KCl, 2.0 mM MgCl_2_, 1.25 mM NaH_2_PO_4_, 26 mM NaHCO_3_, 10 mM glucose, 2.0 mM CaCl_2_ [43]). Oxygenation was accomplished by continually bubbling the solution with 95% O_2_/5% CO_2_.

### 2.3. Drug Treatments

Rats subjected to PVD surgery are injected intraperitoneally with perampanel 3 mg/kg or vehicle control, 1 h after the surgery for 3 consecutive days. Perampanel was freshly dissolved in a solvent mixture of dimethyl sulfoxide (DMSO, Sigma, St. Louis, MO, USA), polyethylene glycol 300 (PEG 300, Sigma, St. Louis, MO, USA), distilled water (1:1:1; *v*/*v*/*v*). The PVD-treated rats with vehicle control are injected with the same solvent mixture without perampanel. Male Sprague–Dawley rats were given perampanel intraperitoneally (i.p.; 3 mg/kg) 1 h after PVD surgery. Perampanel was previously used in different doses, ranging from 1.5 mg/kg body weight to 10 mg/kg body weight [41,44,45,46,47,48]. The dose of 3 mg/kg was chosen based on previous studies and validated by a preliminary experiment on three small-sized groups of rats (sham, PVD, and treatment group, N = 4) based on the therapeutic window of perampanel used in previous studies (1.5–10 mg/kg) to choose the lowest effective dose of perampanel providing neuroprotective effects to avoid any possible toxicity and adverse effects. Following PVD surgery, rats of the treatment group were given a daily dose of perampanel of either 3 mg/kg or 5 mg/kg for three days. After three days, rats were euthanized, and hippocampal slices, from the ipsilateral side of the induced focal cortical ischemia, were stained with PI as described below. The observed neuroprotective effect was the endpoint of the preliminary experiment since the number of hippocampal cells positively stained with PI was a marker of cell death. We found that a 3 mg/kg dose of perampanel was sufficient to prevent hippocampal cell death caused by PVD.

### 2.4. Chemically Induced Long Term Potentiation (cLTP)

Hippocampal slices were submerged in an electrophysiology recording chamber with constant perfusion of oxygenated aCSF (3 mL/min). Field excitatory postsynaptic potentials (fEPSPs) were evoked by orthodromic stimulation of the Schaffer collateral pathway using a bipolar tungsten stimulating electrode (Axon Instruments, Foster City, CA, USA). A pulled glass recording microelectrode filled with aCSF (resistance 1–3 MΩ) was placed in CA1 *stratum radiatum*, which recorded fEPSPs induced by Schaffer collateral stimulation. fEPSPs were evoked for 0.1 ms every 30 s throughout each experiment. The fEPSP signals were amplified 1000 times with an AC amplifier, band-pass filtered at 0.1–100 Hz, digitized at 10 kHz using a Digidata 1440A digitizer (Axon Instruments), and saved to a computer as a Clampex 9.0 (Axon Instruments) file. The collected fEPSP data were analyzed using Clampfit 9.0 (Axon Instruments). The chemically induced long term potentiation experiment consists of three stages; a 10 min baseline recording followed by 10 min of cLTP and a 1 h washout. Baseline recordings were set to approximately 60% of maximal fEPSP values, and a stable baseline was established for 10 min to ensure a stable baseline before inducing cLTP. Following a 10 min baseline, cLTP was induced for 10 min, during which slices were perfused with Forskolin (50 µM, Tocris, Toronto, ON, Canada) and Rolipram (0.1 µM, Tocris) that are dissolved in oxygenated -Mg^2+^ free aCSF. Following cLTP induction, a 1 h washout period in regular (Mg^2+^-containing) aCSF was recorded. The fEPSP slopes were normalized to the mean of the last 10 sweeps (5 min) of the final 10 sweeps (5 min) of the baseline recording period (100%). The mean normalized fEPSP slope was plotted as a function of time with error bars representing the standard error of the mean (SEM). Sample traces are the average of 10 sweeps from a representative recording from each treatment group. All histograms show the mean normalized percent fEPSP of baseline ± SEM. Statistical significance was assessed using one-way analysis of variance (ANOVA) with the Tukey–Kramer post hoc test.

### 2.5. Biochemistry and Western Blotting

To isolate cell surface proteins, slices were treated with NHS-SS-Biotin (1 mg/mL, Thermo Scientific, Waltham, MA, USA) for 1 h at 4 °C. The biotin reaction was quenched with glycine buffer containing 192 mM Glycine and 25 mM Tris (pH 8.3). Slices were then transferred to homogenization tubes and homogenized in lysis buffer (pH 8.0) containing 50 mM Tris, 150 mM NaCl, 1 mM EDTA, 1 mM NaF, and the following protease inhibitors: 1 mM PMSF, 10 µg/mL aprotinin, 10 µg/mL pepstatin A, 10 µg/mL leupeptin, 2 mM Na_3_VO_4_, 20 mM sodium pyrophosphate, 3 mM benzamidine hydrochloride, and 4 mM glycerol 2-phosphate with 1% NP-40 detergent. A Bradford assay was performed with DC protein assay dye (Bio-Rad, Hercules, CA, USA) to determine protein concentration in the lysates, and 500 μg of protein lysate diluted in lysis buffer was loaded into Streptavidin agarose beads (Thermo Scientific) and rotated overnight at 4 °C. The beads were then washed 4–6 times the next day with lysis buffer containing the same reagents as above except 0.1% NP-40. The proteins were eluted by adding 50 µL of 2× Laemmli sample buffer (Bio-Rad) and boiling the samples at 95 °C for 5 min. Whole cell lysate samples of 50 µg were eluted in 20 µL of the same Laemmli buffer and boiled for 5 min. Samples were loaded into 8% SDS-PAGE gels and run for 20 min at 80 V, then the voltage was increased to 160 V and running continued for 1 h. Proteins were transferred from gels to a 0.2 µm PVDF blotting membrane (GE Healthcare Life Sciences, Chicagom IL, USA, 2.5 h, 0.3 mA at 4 °C). Membranes were incubated with 5% fat-free milk for 1 h at room temperature to block nonspecific background and then treated with primary antibodies overnight at 4 °C as follows: GluA1 (rabbit mAb, Millipore, Burlington, MA, USA), GluA2 (mouse mAb, Millipore), GFAP (Proteintech, Rosemont, IL, USA, cat # 16825-1-AP; 1:5000), Iba-1 (Invitrogen, Carlsbad, CA, USA, cat # GT10312; 1:500 dilution), inducible nitric oxide synthase (iNOS, Abcam, Cambridge, UK, Cat # ab3523; 1:1000), and neuronal nitric oxide synthase (nNOS, Millipore, Burlington, MA, USA, cat # 07-571-I; 1:1000). The membranes were reprobed for β-actin (Santa Cruz Biotechnology, Dallas, TX, USA, cat # sc-47778; 1:5000) or GAPDH (Santa Cruz Biotechnology, cat # sc-32233; 1:1000) to normalize levels of proteins. Finally, proteins were visualized by incubating blots with the corresponding HRP-conjugated secondary antibody using enhanced chemiluminescence (ECL) (Biorad), and protein band intensities were quantified using Fiji (NIH, public domain). All data were expressed as the percentage of the intensity of the target protein to that of corresponding loading control.

### 2.6. Propidium Iodide Staining

Propidium iodide (PI) is a fluorescent intercalating agent that is used effectively as marker for cell death and evaluation of cell viability since PI cannot cross the cell membrane of intact healthy cells while only entering and labeling cells with disrupted plasma membranes and producing strong red fluorescence when excited by green light. Propidium iodide staining, and subsequent confocal imaging of rat hippocampal slices were used to examine the effect of perampanel administration on cell survival after ischemic stroke induced by PVD surgery. The methods used were adapted from previous literature [30,31,49,50]. Following equilibration of hippocampal slices for 1 h (see details above), slices were incubated in fresh oxygenated aCSF at room temperature for 2 h prior to a subsequent 1 h incubation with 5 μg/mL propidium iodide (Sigma) in aCSF. Following the incubation period, slices were rinsed thoroughly in aCSF and then fixed in 4% paraformaldehyde at 4 °C overnight. The following day, slices were washed 3 × 15 min in 1× PBS and then mounted on glass microscope slides (VWR) and sealed using Prolong Gold Antifade Reagent (Invitrogen). After the addition of PI, all subsequent procedures were performed in the dark to prevent photobleaching.

Hippocampal slices were imaged using a Zeiss LSM700 laser scanning confocal microscope (Carl Zeiss Group, Toronto, ON, Canada) using green light (543 nm) to induce PI fluorescence. The whole hippocampus was imaged in pieces using a 10× objective lens, and images of CA1 pyramidal neurons were obtained using the Zeiss Plan-Apochromat 63×/1.6 oil objective lens (Carl Zeiss). CA1 images were acquired as Z-stack images of 200 μm depth into the hippocampal slice, with each Z-stack image taken at 2 μm. Two Z-stack images were taken along CA1 for each slice and were averaged using densitometry analysis. Data was collected using Zeiss Zen 2009 version 5.5 software (Carl Zeiss) and was analyzed using ImageJ (Public domain, Version 1.54p). Z-stack images closest to the outer top and bottom of the hippocampal slices were not analyzed, as the neuronal damage in those areas was enhanced by the slicing procedure. The innermost 20 μm (~100 μm into the slice) segments were imaged as z-stacks, and the maximum intensity projections from the different treatment groups were analyzed by using densitometry (Fiji, public domain) and normalizing intensities from treatment groups to their respective sham group with background signal subtracted as previously described [30,31,49,50]. Montages of confocal hippocampal images were combined to show the entire hippocampal slice using Adobe Photoshop CS6 (Adobe Systems, Mountain View, CA, USA).

### 2.7. FluoroJade-C Staining

FluoroJade-C (FJC) is a polyanionic fluorescein derivative that can be used as a sensitive and selective marker for degenerating neurons. Consequently, we used FJC to quantify the level of neurodegeneration in hippocampus after inducing ischemic stroke as described previously [51]. In brief, anesthetized rats were intracardially perfused with 4% PFA in PBS for 30 min, their brains removed and fixed with 4% PFA in PBS overnight, and finally stored in 30% sucrose (*w*/*v*) in 0.1 M PBS for 3 days for cryoprotection before slicing. The frozen brains in liquid nitrogen were then mounted in Tissue-Tek OCT mounting medium, and 40 µm hippocampal slices from coronal sections were obtained with a cryostat. To monitor neurodegeneration, brain sections on gelatin-coated slides were incubated with FJC staining (after air-drying at 45 °C for 20 min) as previously described [51]. Digital images were collected using a Zeiss LSM 700 (Carl Zeiss) with a 10× objective for the hippocampal montages and a 63×/1.4 oil-immersion objective lens for the magnified regions of the hippocampal pyramidal body layers. Two Z-stack images of the CA1 region (taken at 1 μm intervals) were averaged using similar densitometry analysis performed with the PI analysis above. Collected densitometry data was normalized to representative hippocampal slice from the sham group. Data was graphed as a percentage of sham value and analyzed for significance against this control value (100%). Full hippocampal images were assembled as montages of the entire hippocampal slice using Adobe Photoshop CS6 (Adobe Systems, Mountain View, CA, USA).

### 2.8. Pial Vessel Disruption as a Model of Small-Vessel Stroke

Disruption of class II size vessels on the surface of the cortex (pia), known as pial vessel disruption (PVD), has been shown to induce a small focal cortical lesion, which within 3 weeks forms a lacuna-like fluid-filled cyst surrounded by a barrier rich with reactive astrocytes [51,52,53]. This PVD model mimics a small vessel in vivo animal stroke model with several advantages over other stroke models [29,51]. For instance, this PVD model represents a non-perfusion small-vessel stroke model that produces permanent damage to Class II size vessels, and the resulting cortical lesions of approximately 1 mm^3^ are highly reproducible and closely resemble a lacunar infarction [52,54]. In contrast, other focal or global animal stroke models represent ischemic/reperfusion models that involve transient occlusion of large vessels, such as middle cerebral artery occlusion (MCAO) model [55,56], and the ischemic brain damage is often widespread and encompasses greater volume.

The PVD surgery was recently described [33]. In brief, approximately 250 g male Sprague-Dawley rats were anesthetized with 2% isoflurane and kept immobile by a stereotaxic frame. The animal’s body temperature was kept constant throughout the surgery at 37 °C by a temperature-controlled heating pad connected to a rectal thermoprobe. Throughout surgery, the animal was maintained by 2% isoflurane and subcutaneously injected with Buprenorphine (0.035 mg/kg) for pain management. A 5 mm diameter craniotomy was performed on the right and rostral side of the bregma near the coronal and sagittal sutures. Upon exposing the pial vessels after removal of the dura, two medium-sized (class II) pial vessels were mechanically ruptured by fine-tipped forceps, and ice-cold sterile saline was applied until bleeding completely stopped. Disruption of pial vessels was confirmed by visible bleeding. The piece of bone was replaced, and the scalp was then closed with a wound clip. Sham animals received the same treatment with dura removal but no vessel disruption, ensuring that potential confounders such as variations in skull structure or traumatic brain injury–like effects were controlled for across groups. Animals were kept in a cage separately under a warm lamp during the recovery from anesthesia, and thereafter, the animals were returned to their cages. As illustrated in our recent publication [33] (see Supplementary Figure S1), rats were euthanized on day 4 after surgery for postmortem analysis either by intracardiac perfusion (for FJC staining/confocal imaging studies) or by deep anesthesia using halothane followed by decapitation for slicing the hippocampus for electrophysiology studies or biotinylation as described above.

### 2.9. Y-Maze

Rats were acclimatized to the behavior room at least 1 h before starting any behavior tasks. The Y-maze apparatus consists of three Y-shaped arms, each with a rectangular base (45 cm × 12 cm) and a wall height of 35 cm. The animals were video-recorded and subjected to two trials separated by a 90 min interval. During the first trial (acquisition), one arm was blocked (novel arm), and the animal was placed in the start arm with its head facing away from the center of the Y-maze. The animal was free to explore both the start arm and the other open arm (old arm) for 15 min. After the first trial, rats were returned to their cages for a 90 min recovery. During the second trial (retrieval), lasting 5 min, rats were placed in the start arm and allowed to explore all arms, including the novel arm that was previously blocked in first trial. Visual spatial cues on the walls outside the maze were maintained throughout animal exploration in both trials. The video-tracking software, EthoVisionXT (Noldus), was used in a single blinded way to determine the percentage of time spent in each arm during the second 5 min retrieval trial.

### 2.10. Open Field Test

The open-field test apparatus consists of a 56 cm × 56 cm square-shaped base with a 57 cm wall height. The bright light shining directly above the 28 cm × 28 cm center square causes the center square to be a highly exposed area of the field, and the exploratory behaviors of animals were video-recorded. Rats initially placed in the center square were then free to roam the entire open field for 15 min. Rodents are known to explore mainly the peripheral areas of an open field, a phenomenon called thigmotaxis; thus, open field test is accepted as a validated behavioral test to correlate anxiety levels with the amount of thigmotaxis shown by the animal in the open field [57,58]. To determine the percentage of time related to center square entries and total duration spent in center square, EthoVisionXT was used. Also, the heat maps for both Y maze and Open field tasks were generated using EthoVisionXT.

### 2.11. Forced Swim Test

The forced swim test (FST) reflects depressive symptoms such as despair and learned helplessness. When rodents are first immersed in water, the animals swim as vigorously as possible in an attempt to escape a stressful environment [59]. However, the animal eventually reaches a point of helplessness and despair when animals become immobile [59]. We used the FST to test whether the levels of depressive behavior in rats following PVD surgery could be attenuated by perampanel treatment. The FST apparatus is a 30 cm × 30 cm × 60 cm rectangular Plexiglas container with two-thirds filled with water at 25 °C [59]. Rats were allowed to swim for 10 min, and their behavior was recorded by a video camera and analyzed using EthoVisionXT (Version 12, Noldus, Leesburg, VA, USA). To prevent animals from being stressed, which may negatively influence their performance in the other behavioral tests, we reserved the FST as the final behavioral test performed before animals were euthanized the next day.

Rats were scored for both success and vigor as a function of continuous movement of 4 limbs and swimming with head above water, respectively [60], using the following criteria for success and vigor scores as recently described in [33]. Animals were excluded if they spent more than 20 s at the bottom of the tank. Swimming success and vigor scores during the last three minutes of the test were used to evaluate the immobility of rats undergoing different treatments.

### 2.12. Rotarod

The rotarod test was performed as previously described by Deacon [61] to measure motor coordination and post-stroke motor deficits with some modification. The rotarod apparatus (Powermax II, 1.8° step motor, Columbus Instruments/Kollmorgen, Radford, VA, USA) consisted of four chambers. Rats were first acclimatized in the behavioral room 30 min before testing. Rats were pre-trained in the rotarod for 2 days before induction (two trials each day). Then, after 3 days following PVD surgery, rats were placed in each chamber on the rod facing away from the direction of rotation (accelerated rate of 5 rpm/s; maximum time and speed of the rotation at 120 s and 50 rpm, respectively). The test was repeated three times to determine the average latency time of falling from the rod. The individual who scored the latency times was blinded to the treatment groups.

### 2.13. Structural Modeling and Docking

Sequence alignment of rat GluA1 and GluA2 was generated using multiple sequence comparison by log expectation (MUSCLE) using rat *Gria1* (GluA1) (Uniprot ID P19490) and *Gria2* (GluA2) (Uniprot ID P19491) sequences. The structure of rat GluA1 was modeled in 3D using de novo protein trRosetta-constrained energy-minimization protocol [62], and the chemical structure of perampanel was obtained from PubChem (CID: 9924495). The molecular docking study was carried out using the autodock vina module implemented in PyRx tool. The 3D model of *Gria1* was constructed with modeler [63] using *Gria2* as template (PDB: 5L1F). The obtained models were energy minimized, and quality was assessed as described in [64]. Interaction between *Gria1*-Perampanel (PER) was predicted using similarities of superposed protein using the 5L1F structure. Visualization of *Gria1*/*Gria2*-PER models was obtained with PyMOL software (http://pymol.org/, accessed on 25 September 2023).

### 2.14. Enzyme Linked Immunosorbent Assay (EILSA)

Three days post-PVD, animals were euthanized, and hippocampal tissue lysates were subsequently prepared as described above and used to measure the levels of pro-inflammatory and anti-inflammatory mediators (N = 6 animals used from each group). The levels of IL-4, TNF-α, and TGF-β were calculated using commercially available ELISA kits (rat anti-rabbit of each cytokine, Thermofisher Scientific, Waltham, MA, USA). Initial whole cell lysates were prediluted (1:10) based on a trial run to reach the appropriate dilution factor to allow measurements in duplicate to fall within the standard curve of the purchased ELISA kits.

### 2.15. Statistical Analysis

GraphPad Prism 6.0 (GraphPad) was used to construct bar chart summary data with mean ± SEM for all treatment groups. Statistical significance was assessed using a one-way or a two-way ANOVA test as appropriate and a Tukey–Kramer multiple comparison post hoc test with a 95% confidence interval using GraphPad InStat version 9.0 (GraphPad, La Jolla, CA, USA). Two-way ANOVA with Tukey–Kramer multiple comparison test was used to assess statistical significance for ipsilateral and contralateral sides in PI staining, FJC staining, and cLTP recordings. Data normality was tested using the Shapiro–Wilk test prior to statistical analysis. Parametric tests (one-way or two-way ANOVA) were applied to normally distributed datasets. Values were considered statistically significant when the probability values were less than 0.05. Statistical significance was set at *p* < 0.05. For clarity, *p*-values are reported as threshold values (*p* < 0.05, *p* < 0.01, *p* < 0.001). Video recordings from the Y-maze and Open Field Test behavioral tests were analyzed using EthoVisionXT software. Scoring of rotarod and FST behaviors was performed manually in a single blinded manner. Electrophysiological cLTP recordings used random slices from ipsilateral or contralateral hippocampal slices to minimize the length of time of slice preincubation, which could be a possible confounding variable.

## 3. Results

### 3.1. Perampanel Attenuated PVD-Induced Cognitive Dysfunction

**Figure 1:**
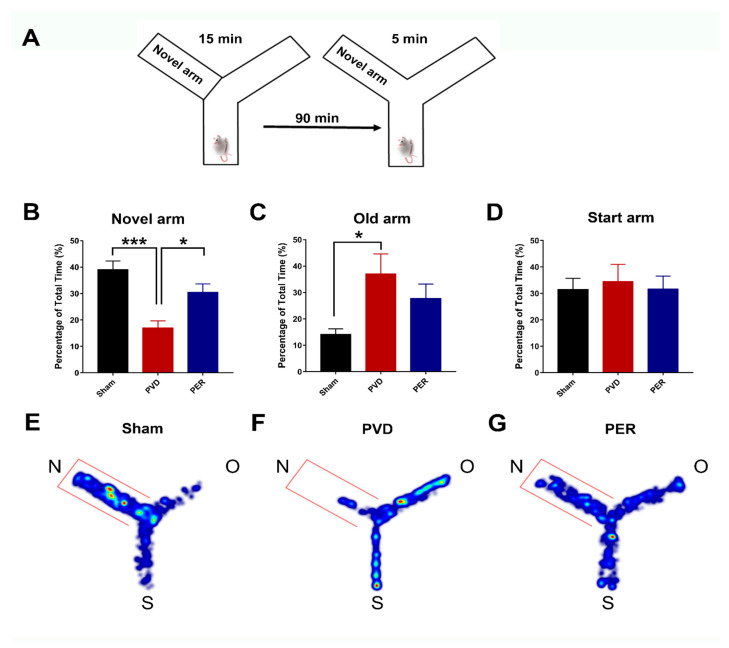
Perampanel restored cognitive function as shown by increased exploration of the novel arm in comparison to PVD-only treated rats. The PVD group displayed the lowest time exploring the novel arm and the highest percentage of time spent in the old arm compared to the other treatment groups. Arm durations were calculated as a percentage of the 5 min second retrieval trial. (**A**) The cartoon figure represents the Y-maze task. (**B**–**D**) Bar graphs represent percentage of time spent in novel, old, and start arms, respectively. (**E**–**G**) Representative heat maps of the 5 min second trial acquired from Ethovision for sham, PVD + vehicle control, and PVD+ perampanel 3 mg/kg, respectively (N = novel arm, O = old arm, and S = start arm of the maze). N values = 11 for each treatment group. Values are shown as mean ± SEM. One-way ANOVA revealed a significant overall group effect (percentage of time spent in novel arm; *p* = 0.0001); post hoc comparisons (Tukey’s test): Significance values: * = *p* < 0.05, *** = *p* < 0.001.

First, we tried to assess the ability of perampanel to improve functional outcomes of ischemic stroke, specifically cognitive impairment; thus, we performed a Y-maze test to evaluate the hippocampal-dependent spatial memory of rats 3 days after PVD surgery. Figure 1 shows that administration of perampanel significantly improved the cognitive deficits in rats subjected to PVD stroke. PVD lesion induced significant cognitive impairment (*p* < 0.001), as demonstrated by the significantly reduced time that the PVD-treated group spent in the novel arm of the Y-maze (16.94 ± 2.70%) compared to the sham group (39.05 ± 3.29%) (Figure 1B). In contrast, the PVD-treated rats spent more time (*p* < 0.05) exploring the old arm (37.02 ± 7.56%) compared to the novel arm of the maze (Figure 1B,C), which indicates failure of rats in PVD group to recognize the novel arm (16.94 ± 2.70%) of the maze, suggesting potential cognitive deficits induced by disrupting pial blood vessels. Interestingly, perampanel successfully helped restore cognitive function after ischemic injury and noticeably prevented PVD-mediated deficits of hippocampal-dependent spatial memory. Therefore, as can be gleaned from the heat maps (Figure 1E–G), the PVD-perampanel treated rats showed significantly improved memory performance as reflected by their significantly increased exploration of the novel arm of the Y-maze (30.47 ± 3.19%) versus the PVD-treated group (16.94 ± 2.70%).

### 3.2. Perampanel Attenuated LTP Deficits in the Ipsilateral Side of Ischemic Lesion

**Figure 2:**
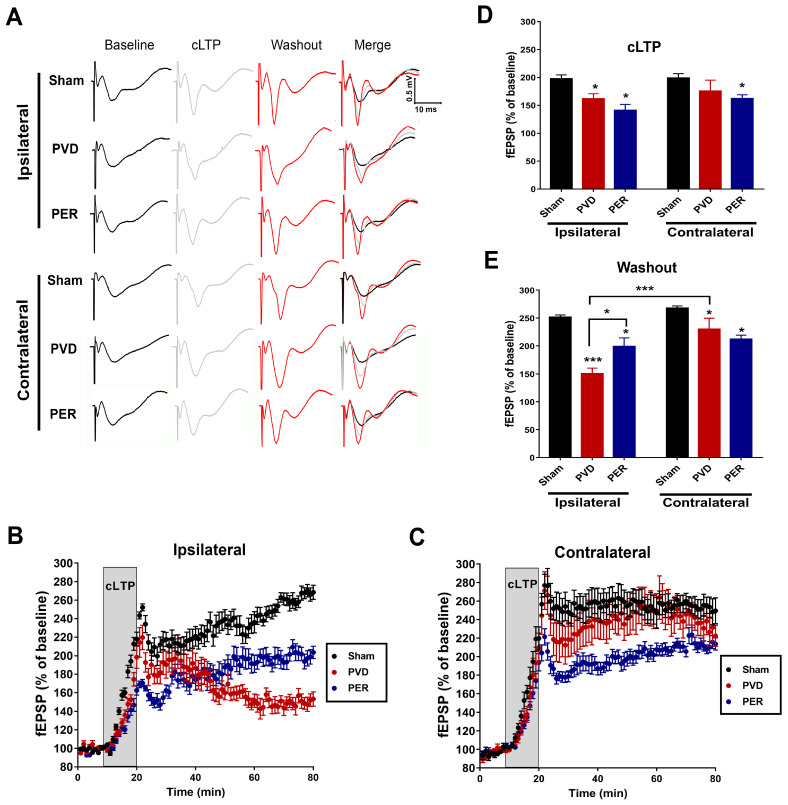
Administration of perampanel inhibited deficits in maintaining cLTP in ipsilateral side of the hippocampus 72 h following PVD. Hippocampal slices of both the ipsilateral and contralateral sides from the perampanel- and vehicle-treated groups were perfused with 50 µM Forskolin and 0.1 µM Rolipram in Mg ^2+^-free aCSF for 10 min to induce long term potentiation (LTP), followed by a 60 min washout to demonstrate the ability to maintain cLTP. (**A**) Representative fEPSP traces showing average sample traces of the last 10 sweeps (final 5 min) of the baseline recording (in black color), 10 min cLTP (in gray color), and then the last 5 min of the 60 min washout period (in red color), and merge of the three traces together from left to right (baseline + cLTP + Washout). Treatment groups from top to bottom are as follows: ipsilateral sham, PVD + vehicle, and PVD + Perampanel, then contralateral sham, PVD + vehicle, and PVD + Perampanel. (**B**,**C**) Time course plots of the average fEPSP slopes, which were normalized to the baseline value (100%) of each recording from ipsilateral and contralateral hippocampal slices, respectively. Sham, PVD + vehicle control, and PVD + perampanel are presented in black, red, and blue colors, respectively. (**D**,**E**) Summary bar graphs showing the mean normalized fEPSP slope percentage of the final 5 min (10 sweeps) of the 10 min cLTP and 60 min washout periods, respectively. Groups are compared to corresponding sham hippocampal slices for significance. N = 6 slices for each treatment group, from different rats. Graphed values show mean ± SEM. Two-way ANOVA revealed a significant main effect of group during the last 5 min of both the cLTP (*p* = 0.0008) and washout periods (*p* = 0.0001). A significant main effect of hemisphere was observed only during the washout phase (*p* = 0.0005; cLTP *p* = 0.1820). The group × hemisphere interaction was significant during washout (*p* = 0.0083) but not during cLTP (*p* = 0.6668). Post hoc comparisons (Tukey’s test): Significance values: * = *p* < 0.05, *** = *p* < 0.001.

Since AMPARs are important in LTP and synaptic plasticity [65,66], we investigated the potential underlying mechanism of perampanel in prevention of PVD-induced cognitive dysfunction and the global effect of ischemic stroke on the process of learning and memory in the brain. We performed fEPSP electrophysiology recordings from freshly prepared ipsilateral and contralateral hippocampal slices after three days of PVD surgery. LTP was chemically induced as described previously by Chen et al. [50]. Briefly, hippocampal slices were perfused with Forskolin (50 µM) and Rolipram (0.1 µM) for 10 min, followed by a 60 min washout to evaluate the ability of neurons to maintain the chemically induced LTP. Focal cortical ischemia induced by PVD lesion caused significant LTP deficits for ipsilateral hippocampal neurons of vehicle control treated rats (150.43 ± 9.95%) compared with the sham group (251.54 ± 1.92%, *p* < 0.001), as shown in Figure 2A,B,D,E. In contrast, contralateral hippocampal neurons of PVD-vehicle control treated rats showed a markedly higher ability to maintain cLTP during the 1 h washout (229.83 ±19.70%, *p* < 0.001) compared to the ipsilateral PVD-treated rats, as shown in Figure 2A,C–E; however, both ipsilateral and contralateral sides of the hippocampus of sham groups showed no significant changes in their ability to maintain the chemically induced LTP (Figure 2). Surprisingly, administration of perampanel did not cause further enhancements of cLTP of the contralateral hippocampal neurons that were maintained during 1 h washout (211.83 ± 7.46%); however, it resulted in insignificant attenuation of cLTP during the washout period (Figure 2A,C,E). On the other hand, perampanel significantly prevented PVD-induced cLTP deficits in the ipsilateral side and showed higher maintenance of cLTP during the 1 h washout period (198.94 ± 15.32%, *p* < 0.05) as shown in Figure 2A,B,E. The induction of cLTP measured at the end of cLTP phase showed significant attenuation of the cLTP with PVD vehicle treatment compared to sham group, and this was observed only in ipsilateral hippocampal slices (Figure 2A–D). Moreover, administration of perampanel caused insignificant lower induction of cLTP in both ipsilateral and contralateral hippocampal slices by 28% and 14%, respectively, compared to the corresponding hippocampal slices from the PVD vehicle group (Figure 2A–D).

### 3.3. Perampanel Inhibited the Depressive-like Behavior of Rats in Forced Swim Test Post Cerebral Ischemia

**Figure 3:**
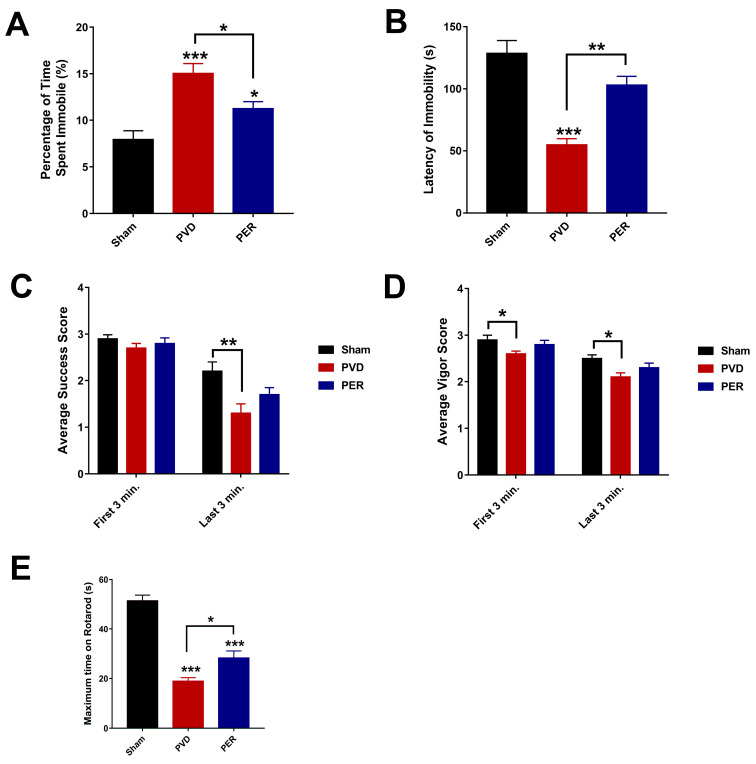
Administration of perampanel improved depressive-like behavior and motor deficits following PVD. Depressive-like behavior was assessed using the forced swim test. Rats were placed in a water tank for 10 min, and time spent immobile was calculated as a percentage of the total trial time. Latency to immobility was plotted as times in seconds. (**A**) The PVD-perampanel treated group improved the immobility and showed a 50% reduction in time spent immobile compared to the vehicle-control group. (**B**) Perampanel treatment showed 100% improvement in latency time of immobility. (**C**,**D**) Average success and vigor scores were measured as mentioned in the methods section. In addition, we used the rotarod test to assess motor deficits 3 days post-PVD. Administration of perampanel attenuated motor deficits caused by PVD ischemic lesion. Rats were pre-trained on the rotarod for 2 days before induction (two trials each day) and then tested 72 h following PVD surgery. (**E**) The bar chart shows the average maximum time spent on the rotarod before falling. Perampanel-treated rats showed significant improvement in the overall motor activity; however, the PVD-vehicle control group spent the least time on the rotarod. N = 12 for each treatment group. Values are shown as mean ± SEM. One-way ANOVA revealed a significant overall group effect in the Rotarod test (*p* = 0.0001) and in the Forced Swim Test (FST) for both percent immobility and latency to immobility (*p* = 0.0001). Two-way ANOVA was used to analyze success and vigor scores of FST. Results showed a significant main effect of group for vigor scores (*p* = 0.0008) and a significant effect of vigor score (first vs. last 3 min, *p* = 0.0001), with no significant group × time interaction (*p* = 0.8071). For success scores, there was a significant main effect of group (*p* = 0.0021) and of first vs. last 3 min (*p* = 0.0001), but no significant group × time interaction (*p* = 0.0712). Post hoc comparisons significance values: * = *p* < 0.05, ** = *p* < 0.01, and *** = *p* < 0.001.

Post-stroke depression is a common health problem that occurs in 30% of stroke survivors [67,68]; thus, the Forced Swim Test (FST) was conducted to better understand the effect of perampanel on the depressive-like behavior in our pre-clinical stroke model. Since the elevated extracellular glutamate was linked to depression, we hypothesized that administration of the AMPAR antagonist perampanel will attenuate depressive-like behavior induced by the ischemic lesion. The PVD-vehicle control treated group showed significantly higher percentage of time spent immobile compared to sham group (*p* < 0.001), indicating a significant depressive-like behavior and learning of helplessness in rats subjected to PVD surgery (Figure 3A,B). In addition, the PVD-vehicle control group showed a significantly lower scores of both success and vigor (Figure 3C,D) in the last 3 min of FST, indicating less ability to use four limbs for continuous movement to float and less time of having their head above water, respectively. These observed results indicate that ischemic lesion induced by PVD surgery caused depressive-like behavior in the affected rats. In contrast, the PVD + perampanel group showed comparable results of latency time for immobility compared to the sham group, indicating a significant improvement in the post-stroke depressive-like behavior observed in the ischemic rats. Moreover, perampanel treatment showed a marked increase in latency of time for immobility (*p* < 0.01) and significantly decreased percentage of time spent immobile (*p* < 0.05) compared to PVD-vehicle control group. Therefore, the observed improvement in both success and vigor scores and the increased latency of immobility in perampanel-treated rats are indicative of an antidepressant action of perampanel.

### 3.4. Perampanel Prevented Post-Stroke Motor Deficits Caused by PVD Lesion

Motor impairments are the most common deficits manifested in stroke patients following recovery [69]. In order to assess motor deficits and motor incoordination in our preclinical in vivo stroke model, we used the rotarod task. We hypothesized that perampanel may attenuate depressed motor activity and the potential impaired motor coordination caused by PVD-ischemic lesion. Indeed, rats subjected to focal ischemic lesion showed significantly less time spent on the rotarod and exhibited 100% less latency time of falling compared to the sham group (*p* < 0.001). Interestingly, perampanel restored the motor activity and significantly improved motor coordination as exhibited by the increased latency time of falling compared to the PVD-vehicle control group, as shown in Figure 3E (*p* < 0.05).

### 3.5. Administration of Perampanel Prevented PVD-Induced Anxiety Like-Behavior

**Figure 4:**
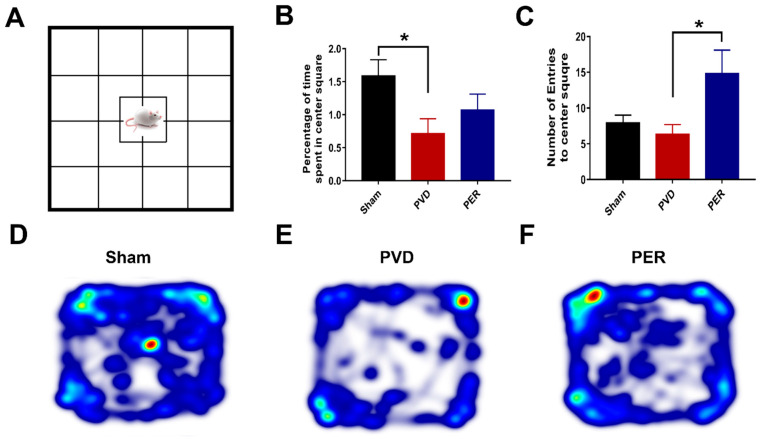
Perampanel attenuated anxiety-like behavior post-PVD. Perampanel increased center square entries while not significantly restoring center square duration in rats subjected to PVD-vehicle control; however, the PVD-vehicle control treated group exhibited the lowest time and minimal entries to center square. (**A**) Schematic representation illustrating the open field task. (**B**) Center square durations were calculated as a percentage of the 15 min trial. (**C**) Number of entries to center square during the task. (**D**–**F**) Representative heat maps from each treatment group were acquired on EthoVision. N = 12 for each treatment group. Values are shown as mean ± SEM. One-way ANOVA revealed a significant overall group effect for both the number of entries into the center square (*p* = 0.0196) and the duration spent in the center square (*p* = 0.0451). Post hoc comparisons significance values: * = *p* < 0.05 vs. sham (Tukey’s test).

Ischemic stroke is associated not only with impaired memory and reduced synaptic plasticity but also with anxiety and phobic disorders that are usually reported with stroke patients after recovery [70]. Therefore, we performed an open field test on the third day after PVD surgery to evaluate the ability of perampanel to prevent post-stroke anxiety-like behavior. Interestingly, we found that focal cortical ischemia induced by PVD lesion significantly increased the signs of anxiety poststroke (*p* < 0.05), as observed by increased thigmotaxic behavior of PVD-vehicle control treated rats, where they spent less percentage of time in the center square of the open field box (0.71 ± 0.23%) and spent more time in the corners (Figure 4B,D,E). In contrast, the sham group spent more time exploring the center square of the open field (1.59 ± 0.25%) compared with the PVD vehicle group, as shown in (Figure 4B,D,E). Surprisingly, administration of perampanel markedly attenuated the increased thigmotaxis behavior and related anxiety observed in PVD rats (Figure 4C,E,F) by increasing the potential to explore the center square of the open field box. As expected, the perampanel-treated group showed a significant (*p* < 0.05) increase in number of entries to center square of the open field (14.83 ± 3.36) compared to the PVD-vehicle control group (6.33 ± 3.28). Moreover, perampanel treatment resulted in moderate elevation in the total percentage of time spent in center square, which indicates the ability of perampanel to prevent post-stroke anxiety-like behavior (Figure 4B).

### 3.6. Perampanel Treatment Partially Prevented PVD-Induced Downregulation of Surface GluA2 While Potentiated Surface Expression of Phosphorylated p-S831 and p-S845 GluA1 in Hippocampus

**Figure 5:**
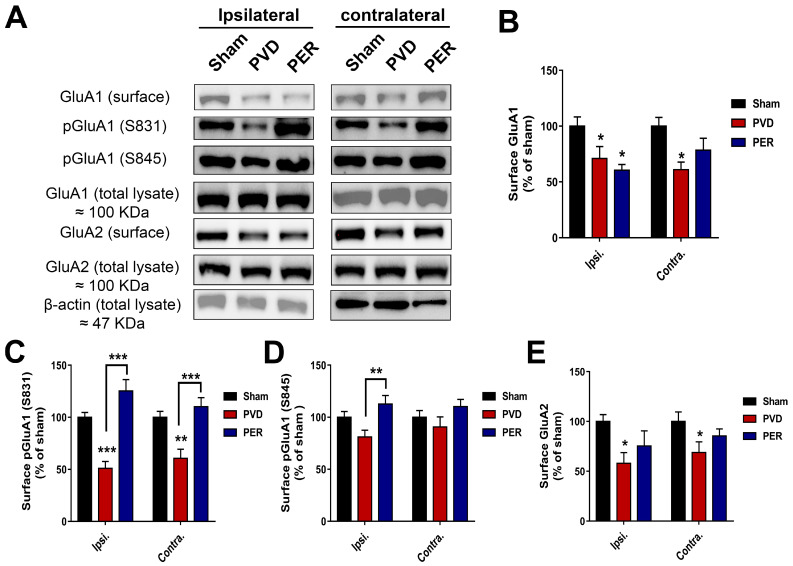
Effect of perampanel on surface levels of GluA1 and GluA2 in both sides of the hippocampus 72 h post-PVD. Focal ischemia resulted in significant decrease in surface levels of both GluA1 and GluA2 on both sides of the hippocampus, and perampanel did not prevent these effects. Total levels of GluA1 and GluA2 were normalized to the corresponding expression of β-actin, and ratios of surface levels/total protein were calculated and expressed as percentage of sham. (**A**) Representative images of Western blots of surface GluA1 and GluA2 from both ipsilateral and contralateral hippocampal tissue lysate. (**B**–**E**) Bar charts showing the quantification of surface GluA1, pGluA1-S831, pGluA1-S845, and surface GluA2, respectively, in both ipsilateral and contralateral sides. Sham (black), PVD-vehicle control (red), and PVD-perampanel treated group (blue). Focal cortical ischemia caused a significant decrease in surface expression of GluA1 and GluA2 AMPAR subunits and reduced the ratio of phosphorylated S831 and S845 of GluA1. Perampanel treatment partially restored the levels of GluA1 AMPAR subunits in contralateral hippocampus and increased the pGluA1-S831 and pGluA1-S845 from both the ipsilateral and contralateral hippocampus. N = 4 from different rats for each treatment group. Graphed values show mean ± SEM. Two-way ANOVA was used to analyze group and hemisphere effects. Overall results showed only a significant difference between treatment groups, with no significant effect of hemispheres ipsilateral vs. contralateral within the group or interaction between group and hemispheres. Surface GluA2 expression showed a significant main effect of group (*p* = 0.0093), with no significant hemisphere effect (*p* = 0.4221) or group × hemisphere interaction (*p* = 0.8451). Surface phosphorylated GluA1 at Ser831 (*p*-S831) exhibited a significant main effect of group (*p* = 0.0001), but no effect of hemisphere (*p* = 0.7757) or interaction (*p* = 0.3031). Similarly, surface phosphorylated GluA1 at Ser845 (*p*-S845) showed a significant group effect (*p* = 0.0094), with no significant hemisphere effect (*p* = 0.6948) or interaction (*p* = 0.6914). Total GluA1 levels also differed significantly between groups (*p* = 0.0014), with no significant hemisphere effect (*p* = 0.7062) or interaction (*p* = 0.2758). Post hoc multiple comparisons significance values: * = *p* < 0.05, ** = *p* < 0.01, *** = *p* < 0.001.

Since we observed cLTP deficits in the PVD treatment group and an improvement of cLTP deficits in the perampanel group (Figure 2), we hypothesized that perampanel attenuates PVD-induced internalization of GluA1 and GluA2 AMPAR subunits. To test our hypothesis, we labeled surface proteins from live hippocampal slices from different treatment groups 72 h after PVD surgery, and then surface levels of GluA1 and GluA2 were determined using biotinylation assays and Western blot analysis. We previously reported that subjecting hippocampal slices to 20 min hypoxia and focal cortical ischemia in our in vivo ischemic stroke model resulted in significant decrease in surface expression of both GluA1 and GluA2-AMPAR subunits 48 h post-PVD [29]. Similarly, our current study shows that PVD lesion induced significant downregulation of surface-expressed GluA1 and GluA2 in both ipsilateral and contralateral sides of the PVD lesion compared to sham group (Figure 5). Interestingly, administration of perampanel partially attenuated the PVD-induced internalization of surface GluA2 in both ipsilateral and contralateral sides (Figure 5A,E). In contrast, perampanel enhanced the total GluA1 internalization in the ipsilateral side and only caused a modest increase in total GluA1 surface expression in the contralateral side (Figure 5A,B). We also evaluated whether perampanel would affect the ratio of phosphorylated GluA1-Ser831 (pGluA1-S831) and GluA1-Ser845 (pGluA1-S845). Indeed, the group subjected to focal cortical ischemia showed a significant 50% and 60% reduction in ratio of pGluA1-S831 in ipsilateral and contralateral hemispheres, respectively, compared to the respective hippocampal tissue from the sham group (ipsi.: *p* < 0.001 and contra.: *p* < 0.01). In contrast, the perampanel-treated rats showed pGluA1-S831 levels comparable to the sham group (Figure 5A,C). In other words, perampanel administration following cerebral ischemia showed a significant increase in level of pGluA1-S831 (*p* < 0.001). On the other hand, the levels of pGluA1-S845 after PVD treatment showed a different pattern from pGluA1-S831, where both ipsilateral and contralateral sides of the hippocampus showed an insignificantly modest reduction in the ratio of pGluA1-S845 compared to sham group (Figure 5A,D). However, perampanel treatment completely restored the ratio of pGluA1-S845 in hippocampus.

### 3.7. Perampanel Binding Domains to GluA1 and GluA2 Subunits

**Figure 6:**
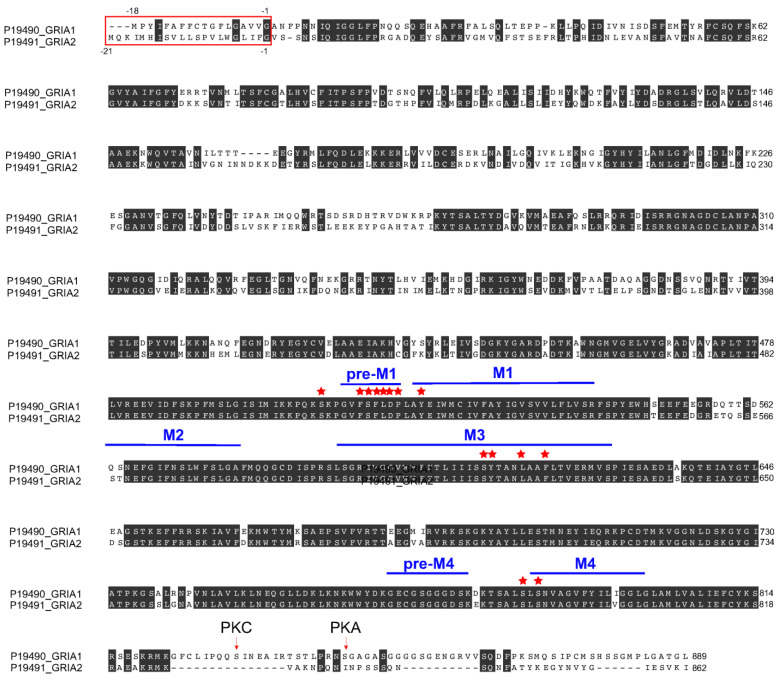
Rat *Gria1* (GluA1) and *Gria2* (GluA2) amino acid sequence alignment. Alignment was generated using multiple sequence comparison by log expectation (MUSCLE) using rat *Gria1* (GluA1) (Uniprot ID P19490) and *Gria2* (GluA2) (Uniprot ID P19491) sequences. Red stars denote amino acid residues that interact with perampanel as revealed from molecular docking analysis (see Figure 7). Amino acid numbers on the right refer to the amino acids of mature protein (excluding the signal sequence in red rectangle). Perampanel binds to a region before transmembrane domain M1 (pre-M1), M1, M3, and M4 regions. Perampanel does not bind to the re-entrant loop M2 domain, the extracellular amino terminal domain (ATD) and ligand binding domain (LBD), or the cytoplasmic domain containing the S831 (PKC site) and S845 (PKA site).

**Figure 7:**
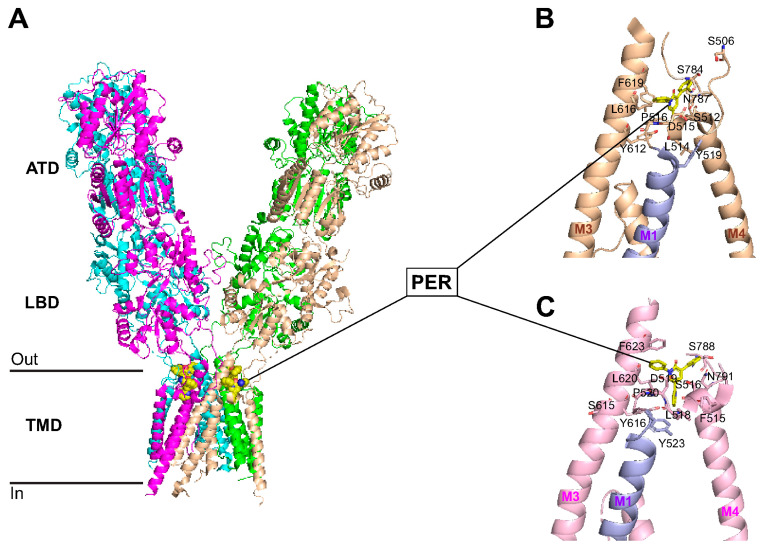
Structure of *Gria1* (GluA1) and *Gria2* (GluA2) interacting with perampanel (PER). (**A**) GluA1 structure viewed parallel to the membrane. Each subunit is in different color. The inner and outer sides of the membrane are indicated by parallel bars. (**B**,**C**) Perampanel is presented in yellow. Close-up views of the binding site in GluA1-PER (**B**) and GluA2-PER (**C**) structures [73] (see text for detail). Hydrogen bonding and hydrophobic interactions with perampanel are described in text. ATD, amino terminal domain; LBD, ligand-binding domain; TMD, transmembrane domain.

We recently studied the effects of multiple AMPAR antagonists in an ex vivo hypoxia/reperfusion ischemic stroke model and showed that perampanel was the only AMPAR antagonist that prevented ischemia-induced neurotoxicity when applied immediately after hypoxic insult [71]. The first 18 amino-terminal amino acids of GluA1, called the signal sequence, are cleaved to give rise to the mature protein, which is 889 amino acids in length [71]. As shown in Figure 6, the two GluA1 phosphorylation sites studied in Figure 5 are located in the C-terminus, namely S831 (a PKC target) and S845 (a PKA target), which, when phosphorylated, both contribute to increased insertion of GluA1 to the surface and increased single channel conductance [72]. The amino acid sequence alignment of Gria1 (GluA1) and Gria2 (GluA2) shows binding of perampanel (PER) to the pre-transmembrane domain M1 (pre-M1) and the transmembrane domains M1, M3, and M4 of GluA1 and GluA2 subunits (amino acids bound by PER indicated with red stars, Figure 6). As shown in Figure 7, PER did not bind to the amino terminal domain (ATD), the ligand binding domain (LBD), or the cytoplasmic domain. Specifically, PER formed hydrogen bonds with S512 and P516 as well as hydrophobic interactions with F511, F513, L514, and D515 in the pre-M1 and hydrophobic interaction with Y519 in M1 region of GluA1 (Figure 7B). An additional hydrophobic interaction between GluA1 and PER occurs at S506-GluA1 preceding the pre-M1 region (see Figure 6 and Figure 7B). Similar PER-binding sites were found in the pre-M1 region of GluA2 (Figure 6 and Figure 7C) (i.e., hydrogen bond with S516, and hydrophobic interactions with F515, F517, L518, D519, P520) and in the M1 domain (Y523), as previously shown by Yelshanskaya and colleagues [73]. PER also formed hydrophobic interaction with the M3 domain of GluA1 (S611, Y612, L616, and F619) and GluA2 (S615, Y616, L620, and F623) (Figure 6 and Figure 7B,C). In addition, PER formed hydrophobic interaction with GluA1 (S784, before M4) and GluA2 (S788, before M4) and a hydrogen bond with GluA1 (N787 in M4) and with GluA2 (N791 in M4, as previously shown by [73]).

### 3.8. Administration of Perampanel Inhibited PVD-Induced Hippocampal Cell Death in Both Ipsilateral and Contralateral Sides

**Figure 8:**
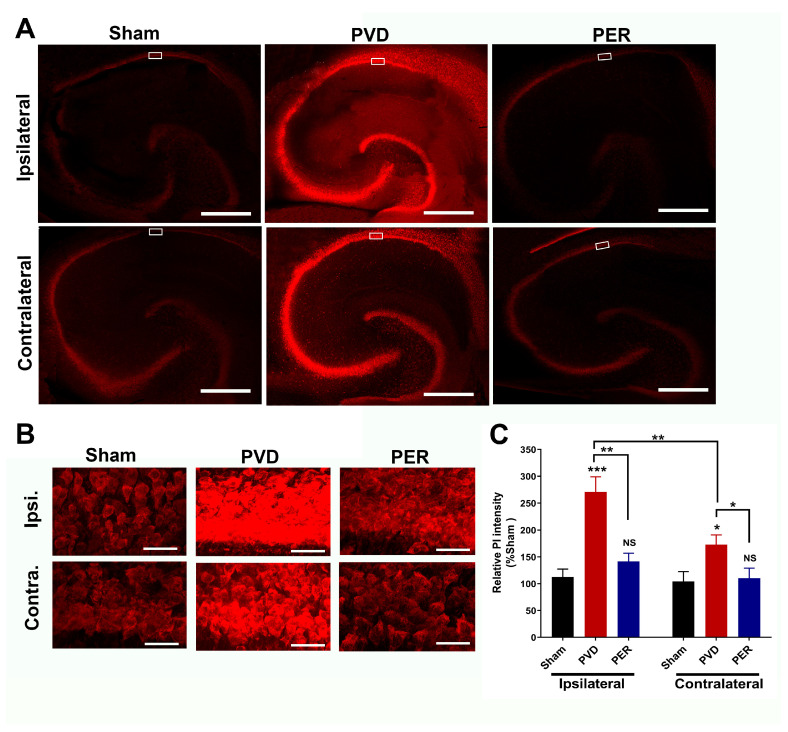
Effect of perampanel on PVD-increased cell death. Administration of perampanel decreased cell death in both ipsilateral and contralateral sides of the hippocampus. Hippocampal slices were stained with propidium iodide (PI), a fluorescent marker for cell death. (**A**) Full montage of hippocampus showing PI fluorescence obtained with 10×. Increased PI fluorescence indicates increased cell death. (**B**) 63× of the representative white boxed regions in (**A**) showing the CA1 area of the hippocampus stained with PI. Scale bars: 1 mm (whole hippocampus, in (**A**)) and 10 µm (CA1, in (**B**)). (**C**) Summary bar graph showing relative PI intensity of the CA1 images in sham, PVD + vehicle control, and PVD + perampanel groups. Perampanel treatment significantly reduced neuronal death in both ipsilateral and contralateral sides of the hippocampus. Levels of hippocampal neuronal damage were lower in contralateral compared to ipsilateral side of the PVD lesion. N = 5 for each treatment group. Values are shown as mean ± SEM. Two-way ANOVA was used to analyze the effects of group and hemisphere. Results showed a significant main effect of group (*p* = 0.0001) and a significant effect of hemisphere ipsilateral vs. contralateral (*p* = 0.0082), with no significant group × hemisphere interaction (*p* = 0.0777). Significance values of post hoc multiple comparisons: * = *p* < 0.05, ** = *p* < 0.01, *** = *p* < 0.001, NS = non-significant.

To determine the ability of perampanel to attenuate cell death mediated by ischemic stroke, ipsilateral and contralateral hippocampal slices were obtained after three days of PVD to perform propidium iodide (PI) staining. Confocal images of PI-stained hippocampal slices showed that PVD caused a marked increase in cell death in CA1, CA2, CA3, and the dentate gyrus regions of the hippocampus in both ipsilateral and contralateral sides, and this cell death was significantly attenuated by perampanel treatment (Figure 8). We quantified cell death in the CA1 region of hippocampus (boxed regions in Figure 8A; see magnified CA1 regions of interest in Figure 8B) by performing densitometry on images of the CA1 pyramidal layer of hippocampus obtained with high magnification z-stacked confocal imaging (Figure 8B). Figure 8C shows that PVD vehicle control-treated rats showed a significant increase in cell death of 178% compared to the corresponding ipsilateral hippocampal slices of the sham group (*p* < 0.0001). Similarly, the PVD vehicle treated rats also showed a marked elevation in cell death of 87% in contralateral hippocampus compared to the corresponding sham contralateral hippocampal slices (*p* = 0.079). In contrast, the observed cell death caused by ischemic injury in the PVD group was largely attenuated in the perampanel-treated group on both the ipsilateral and contralateral sides of the hippocampus (Figure 8B,C, *p* < 0.001, *p* < 0.05, respectively).

### 3.9. Perampanel Attenuated PVD-Induced Neurodegeneration in Hippocampus

**Figure 9:**
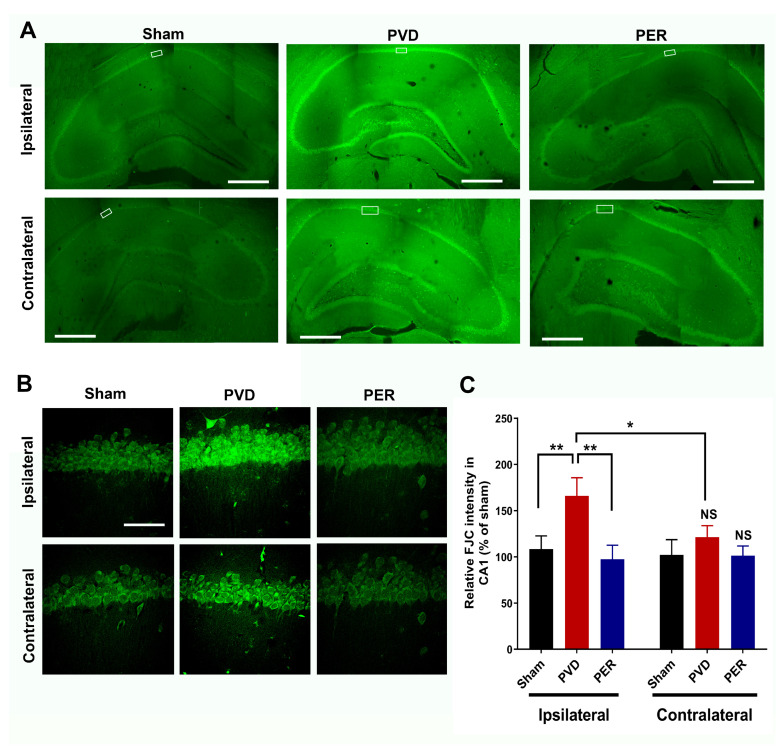
Effect of perampanel on ischemia-induced neurodegeneration. Administration of perampanel attenuated neurodegeneration in hippocampus following PVD. Coronal hippocampal slices were stained with FluoroJade-C (FJC), a specific fluorescent marker for degenerating neurons. (**A**) Full montage of hippocampus fluorescently stained with FJC obtained with 10× objective lens. Full montage images of sham and PVD were adopted from our recent publication [33]. (**B**) 63× magnification of the representative regions of interest in (**A**) (white boxed regions) showing the CA1 area of the ipsilateral and contralateral hippocampus. Scale bars: 1 mm (whole hippocampus, in (**A**)) and 40 µm (CA1, in (**B**)). (**C**) Summary bar graphs of relative FJC intensity of the CA1 images shown in (**B**). N = 5 for each treatment group. Values are shown as mean ± SEM. Two-way ANOVA revealed a significant main effect of group (*p* = 0.0175), while effects of hemisphere (*p* = 0.2211) and group × hemisphere interaction (*p* = 0.2365) were not significant. Significance values of post hoc multiple comparisons: ** = *p* < 0.01, * = *p* < 0.05 NS = not significant.

Next, we assessed PVD-induced neurodegeneration in the hippocampus by quantifying the fluorescence intensities of neurons labeled with FluoroJade-C (FJC), a specific neurodegeneration marker. Figure 9 shows that perampanel treatment completely inhibited the significant neurodegeneration in CA1, CA2, CA3, and dentate gyrus areas of the hippocampal slices of PVD-vehicle control treated rats. Interestingly, the ischemic lesion mediated by PVD surgery resulted in a 70% increase in neurodegeneration of ipsilateral hippocampal neurons in the CA1 area compared to the sham group (*p* < 0.01), which is consistent with the observed cognitive dysfunction and cLTP deficits in PVD-vehicle control treated rats. Moreover, the contralateral side of the PVD-vehicle control showed a modest but insignificant increase in neurodegeneration compared to sham and PVD-perampanel groups (Figure 9C). However, the level of degenerating neurons in the contralateral side was significantly lower (*p* < 0.01) compared to the corresponding ipsilateral side of the PVD-vehicle control group, which agrees with our observations from PI staining mentioned above. Consistent with the effects observed with propidium iodide and FluoroJade-C staining, preliminary data also showed that PVD treatment decreased the number of healthy hippocampal CA1 pyramidal neurons as shown by NeuN labeling compared to sham or PVD plus perampanel treatment group (see Supplementary Figure S3). These results provide proof-of-concept evidence that perampanel can preserve the health of neurons and prevent neurodegeneration mediated by ischemic stroke even if administration was delayed up to 1 h after ischemic injury.

### 3.10. Perampanel Inhibited Neuroinflammation Mediated by Activated Microglia and Astrocytes

**Figure 10:**
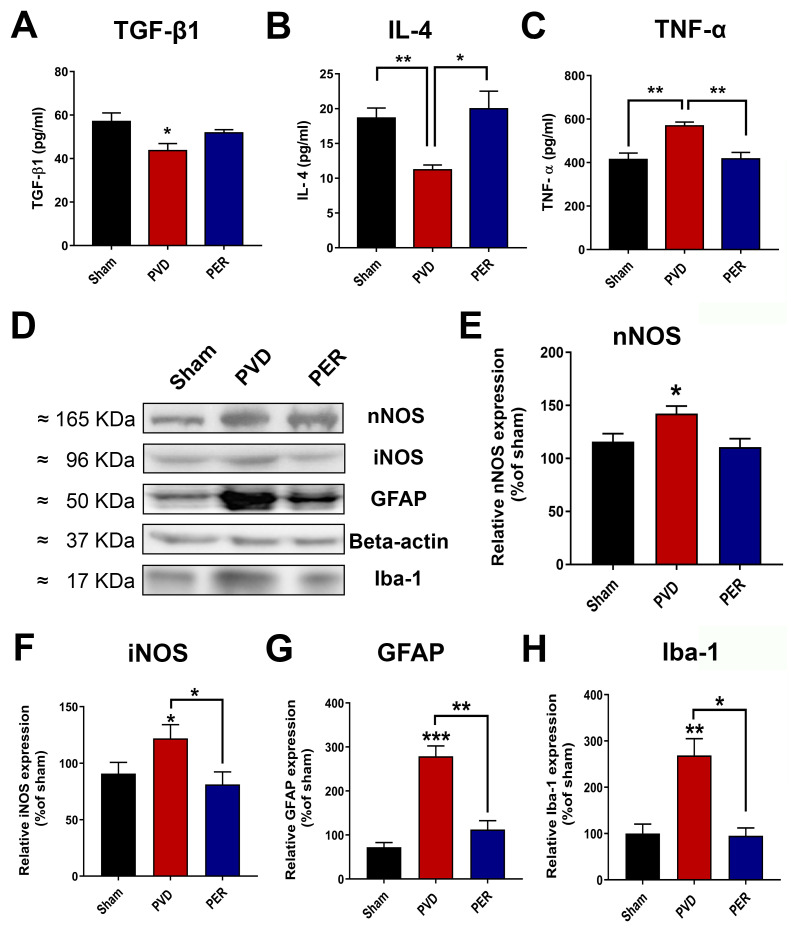
Perampanel oppositely regulates the pro-inflammatory and anti-inflammatory mediators in the hippocampus post-PVD. Perampanel treatment inhibited the PVD-induced elevation of TNF-α, nNOS, and iNOS but recovered the levels of TGF-β1 and IL-4 to near baseline (sham) levels. (**A**–**C**). The total concentration of TGF-β1, IL-4, and TNF-α, respectively, in ipsilateral hippocampal lysates was measured with ELISA kits. Administration of perampanel prevented the increase in nNOS, iNOS, GFAP, and Iba-1 following PVD. (**D**). Representative Western blot images of total ipsilateral hippocampal tissue lysates. (**E**–**H**). Bar chart showing relative nNOS, iNOS, GFAP, and Iba-1 expression, respectively, (% of sham). All tissue lysates were prepared 72 h following PVD surgery. Values are shown as mean ± SEM. N = 6 in each group (independent samples). One-way ANOVA revealed significant overall group effects for IL-4 (*p* = 0.0057), TNF-α (*p* = 0.0011), TGF-β (*p* = 0.0257), nNOS (*p* = 0.0249), iNOS (*p* = 0.0365), GFAP (*p* = 0.0001), and Iba-1 (*p* = 0.0002). Significance values (post hoc Tukey’s test): * = *p* < 0.05, ** = *p* < 0.01, *** = *p* < 0.001.

To determine the role of pro-inflammatory and anti-inflammatory factors in the observed neuronal death in the hippocampus, we measured the concentration of the pro-inflammatory mediators, including tumor necrosis factor-alpha (TNF-α), neuronal nitric oxide synthase (nNOS), and inducible nitric oxide synthase (iNOS), as well as the anti-inflammatory cytokines, including transforming growth factor-beta-1 (TGF-β1) and interleukin-4 (IL-4), in ipsilateral hippocampal lysates. As shown in Figure 10, we found that focal ischemic stroke induced by PVD caused a significant increase in the pro-inflammatory cytokine TNF-α compared to the sham group (N = 6, *p* < 0.01). In addition, both nNOS and iNOS were significantly elevated following cerebral ischemia, suggesting a role for increased nitric oxide production in oxidative stress and cell damage. However, perampanel treatment significantly inhibited ischemic stroke-induced elevation of TNF-α, nNOS, and iNOS (Figure 10C–F). In contrast, the two anti-inflammatory cytokines TGF-β1 and IL-4 in the PVD-vehicle control were markedly reduced by ≈ 23% and 40%, respectively, compared to the sham group (Figure 10A–B). Interestingly, perampanel not only decreased the pro-inflammatory mediators of M1-activated microglia (e.g., TNF-α, iNOS) but also appeared to attenuate the activation of both microglia and astrocytes. The microglia-selective marker Iba-1 (ionized calcium binding adapter molecule 1) and astrocyte marker GFAP (glial fibrillary acidic protein) were significantly elevated after PVD-induced ischemic stroke, and perampanel co-treatment prevented these elevated glial markers (Figure 10D,G,H). These results indicate that the neuroprotective effects of perampanel may, in part, be mediated by its differential regulation of pro-inflammatory and anti-inflammatory markers following stroke that leads to decreased neuroinflammation and post stroke oxidative stress.

## 4. Discussion

### 4.1. Perampanel Binds to GluA1 and GluA2 to Promote Neuroprotection in Pial Vessel Disruption Stroke Model

Elevation of extracellular glutamate following ischemic injury has been recognized as one of the major mechanisms mediating stroke-induced neurodegeneration and cognitive dysfunction. Previously, glutamate excitotoxicity was believed to be mainly mediated by NMDARs, and this has been validated in preclinical trials by the observed neuroprotective effects of NMDAR antagonists, including Selfotel, Eliprodil, and Aptiganel [74]. However, clinical studies of NMDAR antagonists for stroke therapy have thus far been disappointing owing to lack of clinical efficacy or serious psychomimetic adverse effects such as hallucinations and cognitive dysfunction [75]. The present study explored the potential neuroprotective effects of perampanel, a clinically approved non-competitive AMPAR antagonist, in our non-reperfusion ischemic stroke model. We demonstrated that intraperitoneal administration of perampanel 1 h post-stroke attenuated hippocampal neurodegeneration and improved stroke-induced behavioral deficits, which are consistent with the perampanel-induced improvements in synaptic plasticity and adaptations in surface-expressed AMPARs that promote neuroprotection.

Post-stroke cognitive dysfunction is one of the major disability complications in stroke patients after recovery from ischemic attack [76]. Numerous studies have shown impairment of hippocampal-dependent spatial memory with ischemic/reperfusion animal stroke models such as middle cerebral artery occlusion (MCAO) [77,78,79]. Our results using the non-reperfusion PVD ischemic stroke model showed that administration of perampanel shortly after the onset of focal cortical ischemia significantly attenuated the hippocampal-dependent spatial memory deficits. In agreement with our results, other AMPAR antagonists such as GYKI-52466 and NBQX had been successful in prevention of deficits in cognitive function in ischemic/reperfusion stroke models, including bilateral carotid occlusion [80] and four vessel occlusion [81]. However, the non-competitive AMPAR blocker GYKI-52466 did not attenuate impairment in hippocampal-dependent spatial memory if administration was delayed after the induction of cerebral ischemia [81]. In contrast, the present study and previous findings by others [45,46] similarly showed that perampanel had an extended therapeutic window and prevented the cognitive deficits even if administration was delayed after induction of ischemia/reperfusion. Results from our molecular docking analysis showed that the conserved amino acid residues, which were previously reported by others [73] to be involved in perampanel and GluA2 interactions, may also mediate binding between perampanel and GluA1. Specifically, hydrogen bonding existed between perampanel and S512 and P516 of the GluA1 pre-M1 region as well as N787 of the GluA1 M4 region. Yelshanskaya and colleagues [73] found perampanel forming hydrogen bonding at the equivalent site N791 in GluA2 M4 domain as well as additional hydrogen bonds with S516 in pre-M1 segment and Y616 in the M3 domain. Additional hydrophobic interactions were found between perampanel and GluA1 and GluA2 pre-M1, M3, and M4 segments and before the pre-M1 sequence. These structural results are consistent with perampanel acting as a non-competitive antagonist that did not interfere with the ligand-binding domain. Moreover, we recently reported that perampanel, but not other calcium-permeable AMPAR antagonists (e.g., philanthotoxin-74, IEM 1460), significantly inhibited hippocampal neuronal cell death and post-hypoxia synaptic potentiation if administered either 5 min after hypoxia administration or after 45 min of normoxic washout following a 20 min hypoxia [31]. In addition, our present results from the in vivo focal cortical non-reperfusion ischemic stroke model provided evidence that perampanel can also prevent impaired learning and memory associated with permanent ischemia. Previous reports also showed that perampanel effectively attenuated cognitive deficits in animal models of traumatic brain injury (TBI) [48] and status epilepticus (SE) [47], suggesting that this non-competitive AMPAR antagonist has a broad spectrum of neuroprotection against cognitive deficits caused by other neurological disorders.

### 4.2. Perampanel Attenuates Behavioral and LTP Deficits in Pial Vessel Disruption Stroke Model by Increasing pSer845/pSer831 GluA1

Motor deficits and mood dysfunction also represent major complications following stroke [69]. Our results showed that the focal cortical ischemia caused severe motor deficits, similar to those previously reported in MCAO model [82], and that perampanel administration shortly after the onset of cerebral ischemia significantly improved motor activity by ≈40% in the rotarod task. Similarly, the anti-seizure medication valproic acid also improved motor activity of rats following MCAO [83]. Since perampanel was previously shown to cause dose-dependent motor deficits in rats at higher doses (e.g., 9.14 mg/kg) [84] our studies used a lower dose of perampanel (i.e., 3 mg/kg) to minimize any perampanel-induced motor deficit at higher doses. Using 3 mg/kg perampanel, we also showed that perampanel was effective in attenuating ischemia-induced anxiety-like and depressive-like behaviors in rats 72 h following the PVD surgery. Our observation of the decreased thigmotaxis behavior with perampanel treatment in PVD-treated rats suggests that blocking AMPARs by perampanel can help ameliorate post-stroke anxiety that is often associated with stroke patients after recovery [70]. Similarly, the observed PVD-induced anxiety-like behavior has also been reported previously in other stroke models in mice [85]. This post-stroke anxiety-like behavior observed with PVD-treated rats might be induced by elevated extracellular adenosine and glutamate following ischemic stroke [17,26]. We have previously reported that prolonged A1R activation during hypoxia results in clathrin-mediated internalization of GluA1 AMPARs, which accompanies the reduced levels of pGluA1-S831 and pGluA1-S845 in rat hippocampus [50]. Our present result showing PVD-induced reduction in pGluA1-S831 and a previous report by others showing the reduction in pGluA1-S845 in dorsal hippocampus after treatments with anxiolytic drugs [86], indicate an important role of GluA1 phosphorylation in anxiety-related behaviors. Moreover, our findings of decreased anxiety-like behavior in perampanel-treated rats in our PVD stroke model are in agreement with a previous study showing that other non-competitive AMPAR antagonists such as GYKI-52466 can profoundly block anxiety-like behavior in rodents [87]. Interestingly, our results showed that not only anxiety, but also depressive-like behavior was markedly attenuated by perampanel. The observed increase in latency time of immobility and the lower time spent immobile in perampanel-treated PVD rats confirm the observed improvement in motor activity in the rotarod task. In addition, the observed improvements in success and vigor scores in the FST suggest that perampanel can inhibit the depressive-like behavior following cerebral ischemia. Thus, the preserved motor activity with the administration of perampanel may account for, in part, the observed improvement in performance in FST. In contrast, a previous study showed that the antidepressant action of selective serotonin reuptake inhibitors (SSRIs) and tricyclic antidepressants (TCAs) was enhanced by AMPAR potentiators; however, antagonizing AMPAR did not influence their action [88]. Nevertheless, further studies in preclinical stroke models are needed to explore the effect of short-term and long-term use of perampanel on anxiety and depressive behavior following cerebral ischemia. Since perampanel attenuated all the behavioral deficits described above, further studies are also needed to investigate whether perampanel exhibits neuroprotective effects in other brain regions that control mood and motor function.

Consistent with our observation of perampanel-induced improvement in cognitive function after focal cortical ischemia, the present study also demonstrated that perampanel administered after cerebral ischemia significantly improved LTP maintenance in ipsilateral hippocampus; however, perampanel did not improve deficits in LTP induction. Moreover, while LTP induction and maintenance were moderately reduced in contralateral hippocampus compared to the ipsilateral side, perampanel did not appear to improve these PVD-induced LTP deficits. In contrast, other studies using different animal stroke models have reported similar levels of LTP deficits in hippocampus from both brain hemispheres [89,90], but the effects of perampanel on LTP deficits in these stroke models have not yet been reported. Therefore, our study provides evidence that perampanel exhibits a neuroprotective effect in our ischemic/non-reperfusion stroke model by preventing LTP deficits and preserving synaptic plasticity of hippocampal neurons in ipsilateral side of the hippocampus. However, further studies are needed to investigate whether these neuroprotective effects of perampanel and improvements in synaptic plasticity persist in the long term (i.e., weeks to months after cessation of perampanel treatments). Adenosine signaling and AMPAR trafficking can play a very important role in stroke-mediated LTP deficits, as we previously reported that LTP deficit in aged rats was mediated by an A1R-dependent mechanism involving clathrin-mediated endocytosis of GluA1 and GluA2 AMPAR subunits [50].

It is noteworthy that in the contralateral hippocampus, perampanel administration appeared to reduce cLTP relative to sham controls, despite the absence of an effect of PVD alone. This raises the possibility that perampanel may directly influence baseline synaptic plasticity and, by extension, learning and memory processes independent of ischemic injury. Although the present study was not designed to evaluate perampanel’s effects under non-ischemic conditions, and we therefore did not include a perampanel-only cohort, this represents an important limitation. Future studies will aim to incorporate a perampanel-only group to disentangle baseline drug effects from ischemia-related changes in synaptic function.

Despite the ability of perampanel to block AMPARs and prevent LTP deficits and behavioral abnormalities in animal models of cerebral ischemia, TBI, and SE as described above, the cellular mechanisms by which perampanel mediated these neuroprotective effects are not yet well elucidated. Thus, we investigated the changes in surface levels of GluA1 and GluA2 AMPAR subunits, since changes in GluA2 surface levels would affect calcium permeability of AMPARs [91]. We found that focal cortical ischemia mediated reduction in surface levels of both GluA1 and GluA2 subunits 72 h post-PVD, indicating decreased functionality of AMPARs resulting in impaired learning and LTP deficits. Consistent with these in vivo findings, we also previously reported that hypoxia caused downregulation of GluA1 and GluA2 surface expression, which involved clathrin-mediated AMPAR internalization [29,50]. In the present study, we showed that perampanel treatment partially restored the surface levels of GluA2 in both the ipsilateral and contralateral side of the hippocampus, which is expected to lead to the expression of more calcium-impermeable AMPARs and, hence, decreased neuronal damage, as shown in this study. In contrast, perampanel did not attenuate the downregulation of surface-expressed GluA1 subunits of AMPARs ipsilaterally, but instead enhanced the GluA1 surface downregulation. Similarly, perampanel was previously shown to enhance GluA1 internalization following SE [44]. This also suggests that perampanel may promote less expression of calcium-permeable AMPARs in ipsilateral side of the ischemic lesion, which is expected to lead to less calcium excitotoxicity and subsequently to reduced neurodegeneration. Consistent with this, we observed that perampanel promoted neuroprotection in both the ipsilateral and contralateral sides of hippocampus, suggesting that perampanel may have other effects on GluA1 and GluA2 AMPAR trafficking that promote increased neuronal activity and neuroprotection. It is noteworthy that the restored ratio of p-GluA1-S831 and p-GluA1-S845 may reflect the observed improvement in cognitive and anxiety-like behaviors after perampanel treatment in addition to the elevated maintenance of LTP, since the phosphorylated S831 and S845 residues of GluA1 subunits are known to play a crucial role in LTP and synaptic plasticity [92]. Specifically, since perampanel has been shown in normal and rat models of SE to alter the upstream regulators of GluA1 phosphorylation at S831 and S845, including the protein kinases such as Ca^2+^/CaM-dependent protein kinase II (CaMKII), protein kinase C (PKC), cAMP-dependent protein kinase (PKA3), and protein kinase A (PKA) [44]; therefore, further research is needed to reveal the potential of perampanel as a modulator of these protein kinases during cerebral ischemia.

### 4.3. Perampanel Decreased Microglia/Astrocyte Activation and Neuroinflammation in Pial Vessel Disruption Stroke Model

Tissue necrosis and cell apoptosis, mediated by glutamate excitotoxicity, are commonly observed after focal cerebral ischemia and hypoxia in well-established animal stroke models as well as in patients with cerebral infarction [24]. We previously showed that focal ischemic lesion caused by PVD resulted in hippocampal cell death in the ipsilateral side of the hippocampus 48 h following the PVD [29]. This hippocampal neurodegeneration may involve activation of glial cells, consistent with our previous report that the PVD-induced cortical lesions were accompanied by increased microglia and astrocyte density at the injury site, and this increase in glial density was attenuated by minocycline and batimastat, which are known to reduce microglia activation [51]. Notably, the observed improvement in cognitive deficits following focal ischemia in perampanel-treated rats can be explained by the markedly attenuated hippocampal cell death and neurodegeneration as observed with the decreased PI and FJC staining in both ipsilateral and contralateral hippocampal brain slices. Moreover, we showed recently that perampanel prevented hippocampal cell death by inhibiting the AMPAR-mediated adenosine-induced post-hypoxia synaptic potentiation following 20 min of hypoxia [31]. This form of hypoxia-induced synaptic potentiation was dependent on the activities of both A1Rs and A2ARs, which promoted the expression of more calcium-permeable AMPARs that likely contributed to the perampanel-sensitive neuronal damage and neurodegeneration observed in the current study. Likewise, perampanel showed similar neuroprotective properties in global ischemic stroke models, SE, and TBI [45,46,47,48]. Our current findings suggest that perampanel affects AMPARs not only expressed on neurons but also on glial cells, which results in significant attenuation of post-stroke behavioral deficits.

Furthermore, we have examined the potential role of perampanel in suppressing the neuroinflammation induced by glutamate excitotoxicity. Several studies showed the complex role of microglia in neuronal damage mediated by ischemic injury [93,94,95]. Microglial activation is usually classified into two categories, M1 (classical) and M2 (alternate), which lead to promotion or attenuation of inflammation, respectively [96]. For instance, increased density of M1-activated microglia is associated with elevation of pro-inflammatory mediators, including TNF-α and NO. In contrast, M2 phenotypes suppress inflammatory response by secretion of anti-inflammatory mediators such as TGF-β1 and IL-4 [93,94,95,96]. Previous reports from animal stroke models showed that TNF-α potentiates glutamate-induced neuronal damage following cerebral ischemia [97,98,99]. Consistent with these studies, we found that the elevated levels of TNF-α, iNOS, and nNOS post-PVD were attenuated by perampanel treatment, suggesting that blocking AMPARs with perampanel attenuates the release of pro-inflammatory mediators from M1-phenotype microglia or activated astrocytes in cerebral ischemia [100]. Furthermore, our results showed that perampanel attenuated the elevation of glial markers GFAP and Iba-1. Whether AMPARs expressed on microglia or astrocyte surface membranes contribute to cytokine release in our PVD stroke model remains to be established. In contrast to the pro-inflammatory cytokines, our ELISA results showed that perampanel administration following focal ischemia restored the levels of the anti-inflammatory markers TGF-β1 and IL-4. Likewise, perampanel showed similar results in animal models of global ischemic stroke [45,46] and traumatic brain injury [48,101]. The oxidative stress accompanying the changes in these pro-inflammatory and anti-inflammatory mediators was also seen in clinical studies of stroke patients. It will be important in future studies to investigate the neuroprotective role of perampanel in suppressing neuroinflammation in the brains of stroke patients [102,103,104]. Therefore, the repurposing of perampanel for stroke therapy and for other neurodegenerative disorders could significantly attenuate the chronic glutamate toxicity and functional deficits in progressive neurodegenerative conditions. Notably, perampanel has also demonstrated neuroprotective effects in the photothrombotic ischemic stroke model, where it reduced neuroinflammation and oxidative stress by suppressing pro-inflammatory cytokine expression and promoting M2-type microglial activation [105].

### 4.4. Advantages and Limitations of PVD Compared to Photothrombosis and MCAO Models

Unlike the middle cerebral artery occlusion (MCAO) model, which produces large reperfused infarcts, or photothrombosis, which generates highly reproducible but artificially induced lesions via light–dye interactions, the pial vessel disruption (PVD) model replicates focal ischemia without reperfusion through direct mechanical injury of cortical pial vessels. This approach results in lower mortality, avoids widespread microvascular phototoxicity, and more closely mimics clinically relevant ischemia non-reperfusion and vascular trauma.

The photothrombotic stroke model is designed to generate localized ischemic injury in a targeted cortical region following injection of a photosensitive dye. When the area is illuminated, the dye produces reactive oxygen species, which injure endothelial cells. This damage triggers platelet aggregation and thrombus formation, ultimately blocking local blood flow [106,107,108]. However, the mechanism of vascular injury in photothrombosis is artificial, relying on chemical-light interactions that result in widespread endothelial damage and occlusion of capillary networks, which does not closely mimic the mechanical vascular trauma or ischemia without reperfusion frequently observed in human patients. By contrast, the PVD model involves direct mechanical rupture of medium-sized (class II) pial vessels, resulting in localized ischemia non-reperfusion with delayed neurodegeneration and neuroinflammation.

Even though the MCAO model is considered the gold standard ischemic stroke model, there are some challenges that must be taken into consideration. First, MCAO may be complicated by subarachnoid hemorrhage; researchers reported noticeable differences in incidences of certain complications dependent on individual variation between rodents following the MCAO procedure [109]. For instance, transection of the external carotid artery may result in ischemia of muscles of mastication and swallowing, leading to dysphagia and significant weight loss and delayed recovery in 50% of animals, which may affect post-stroke behavioral outcomes [110,111]. Second, ischemia of the hypothalamus, which occurs in permanent MCAO or transient MCAO longer than 60 min, results in thermal dysregulation and post-stroke hyperthermia in rats that lasts for at least one day after surgery [112]. Conversely, human strokes are rarely associated with hypothalamic ischemia [113]. These temperature fluctuations are often not monitored in rats; in addition, hyperthermic response following MCAO leads to exacerbation of cell death and may be a source of variability in cell death in the MCAO model [112,114]. In fact, the primary neuroprotective effect of the NMDA receptor antagonist MK-801 was found to be mediated through its hypothermic effect, and not through prevention of NMDA-induced excitotoxicity in the infarct lesion [115]. Third, the MCAO model might be associated with ipsilateral retinal injury and subsequent visual impairment, which may confound behavioral assessments post-stroke, such as novel object recognition and Y-maze tasks for memory assessment [116]. Fourth, reproducibility of infarction in MCAO is affected by different factors, such as suture diameter, length of filament insertion, type of filament coating, duration of occlusion, experience, and microsurgical skills of trainees performing the MCAO procedure [117].

Previous studies showed that the MCAO model exhibits significant variations in lesion size and post-stroke outcomes not only within the same species but also between different species and strains. MCAO in spontaneously hypertensive rats (SHRs) results in relatively large infarcts, with low variation in size [117]. In contrast, Sprague–Dawley rats, the most commonly used strain in stroke neuroprotective research, exhibit small infarct volumes with considerable variability [117]. Our PVD model induces cortical microvascular injury and secondary neurodegeneration without reperfusion, which more closely mimics clinical cases of non-reperfused stroke than the commonly used MCAO model. This allowed us to examine perampanel’s neuroprotective effects in a pathophysiologically distinct and clinically important context. Unlike MCAO, which causes extensive infarction with high mortality and morbidity, the PVD model produces smaller, more localized infarcts with significantly reduced animal fatality.

### 4.5. Limitations of the Study

Our modified PVD stroke model, which involves disruption of medium-sized pial vessels, mimics a small vessel stroke with a small ischemic cortical lesion that does not extend to the corpus callosum [51]. Within 24 h after PVD, there is initially a breach in the blood–brain barrier and a cavity [52], and within the following 24 h, the lesion is repopulated with cellular material (e.g., microglia, which is likely to produce peak MMP-2 and MMP-9 levels at this time point) [118]. As discussed in our previous studies [51,52,54,118], our modified PVD model after 3 weeks results in the highly reproducible cone-shaped cortical lesion surrounded by glial scar that resembles a lacunar infarction (about 1 mm^3^ volume, no variation in lesion size or shape), and drugs known to inhibit microglia activation and MMP-9 secretion (e.g., minocycline and batimastat) are able to reduce this lesion volume. However, a major limitation of the present study is that we did not assess the effects of perampanel after 72 h post PVD to test whether perampanel would affect microglia invasion of the injury site, but based on the ability of perampanel to inhibit microglia marker Iba-1 and microglia-derived inflammatory/oxidative stress markers (TNF-alpha, iNOS) in the hippocampus 3 days post PVD, we predict that perampanel may contribute to inhibiting microglia hyper-activation at the injury site and ultimately reducing ischemic lesion volume observed at 3 weeks post PVD. Therefore, future studies are needed to determine whether perampanel also reduces the size of the cortical stroke/penumbra (e.g., using Evans blue staining) 3 days after PVD lesion, and whether this reduction in lesion volume and penumbra infarct size directly reflects the reduced secondary neurodegeneration observed in the hippocampus and overall improved animal behavior.

## 5. Conclusions

**Figure 11:**
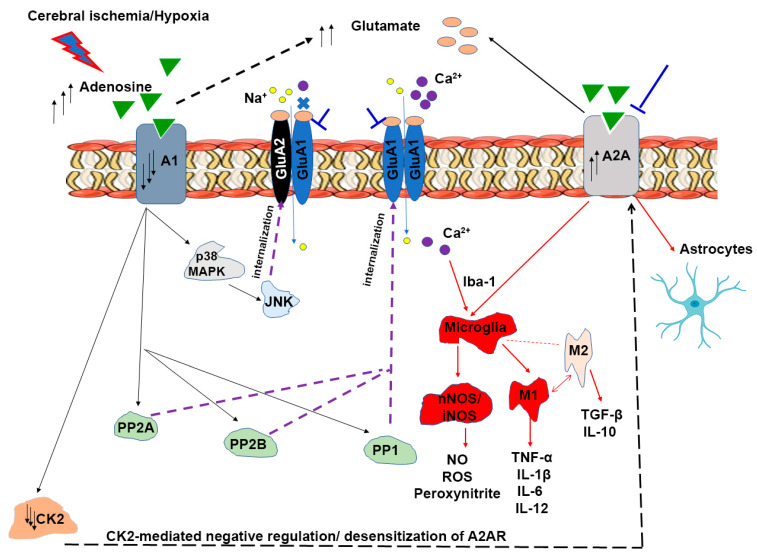
A summary of identified underlying downstream signaling of adenosine A1/A2A receptor crosstalk and regulation of GluA2-lacking AMPA receptors following cerebral ischemia. A1R stimulation leads to clathrin-mediated endocytosis of GluA1 and GluA2 AMPARs via activation of p38-mitogen-activated protein kinase (p38-MAPK), c-Jun N-terminal kinase (JNK), and protein phosphatases PP2A, PP2B, and PP1 [29,30,43,50,67]. Chronic A1R stimulation in ex vivo hypoxia/normoxia ischemia model or a pial vessel disruption (PVD)-induced focal cortical stroke model leads to desensitization of A1R and upregulation of A2AR via casein kinase 2 (CK2) [29,31]. Inhibition of A2AR with istradefylline in PVD-induced stroke model prevents neurodegeneration and neuroinflammation and attenuates behavioral abnormalities [33]. Similarly, inhibition of AMPARs with perampanel in PVD-induced stroke model prevents neurodegeneration and neuroinflammation and significantly reduces LTP and behavioral deficits (this study). Whether combined perampanel and istradefylline treatment produces greater neuroprotection and improved behavioral outcomes in stroke model warrants further investigation. Solid black arrows indicate activation, while purple and black dashed arrows represent endocytosis and desensitization, respectively.

This study provides greater insight into the neuroprotective effects of the clinically relevant AMPAR antagonist perampanel in cerebral ischemia. Our findings demonstrate that perampanel markedly attenuated behavioral deficits, LTP deficits, neurodegeneration, and neuroinflammatory markers 72 h following PVD ischemic lesion. Moreover, perampanel prevented the downregulation of surface-expressed GluA1 and GluA2 AMPAR subunits during focal cortical ischemia. These results are comparable to those previously observed with istradefylline, a selective A2AR antagonist, as reported in our recent publication [33]. In particular, istradefylline similarly attenuated neuronal cell death and neuroinflammation following cerebral ischemia, as well as improved cognitive and motor outcomes in rodent models. This suggests that targeting excitotoxicity through AMPA receptor blockade and modulating neuroinflammatory pathways via A2AR receptor antagonism may represent complementary or converging strategies for mitigating ischemic brain injury (see Figure 11). These findings provide further support for the therapeutic potential of glutamatergic and purinergic receptor modulation in the context of ischemic stroke. Therefore, future studies are needed to determine whether a combinatorial therapy involving the AMPAR antagonist perampanel and the A2AR blocker istradefylline proves more efficacious than monotherapy to reduce neuroinflammation, neurodegeneration, and behavioral deficits in cerebral ischemia. A key limitation of this study is that it remains unclear whether the neuroprotective effects of perampanel are mediated directly through AMPAR antagonism or indirectly via interactions with adenosine signaling pathways and whether longer-term treatment with perampanel in preclinical stroke models will reveal deleterious side effects that might limit its therapeutic utility as a neuroprotective agent in stroke.

## Data Availability

The data that support the findings of this study are available upon request from the corresponding author.

## Supplementary Materials

The following supporting information can be downloaded at: https://www.mdpi.com/article/10.3390/cells14201628/s1, Figure S1: Experimental plan involving ischemic stroke induction with pial vessel disruption (PVD) protocol and treatment groups (vehicle control vs. perampanel, given intraperitoneally) to investigate the potential neuroprotective effects of perampanel; Figure S2: Representative images for Figure 8 (propidium iodide) and Figure 9 (FluoroJade-C); Figure S3: Pilot study showing changes in NeuN (red), nNOS (green), and DAPI (blue) staining in rats subjected to PVD, sham control, and PVD + perampanel.

## Abbreviations

The following abbreviations are used in this manuscript:

PVD	Pial Vessel Disruption
PER	Perampanel
cLTP	Chemically induced long term potentiation
fEPSP	Field excitatory postsynaptic potential
AMPAR	α-amino-3-hydroxy-5-methyl-4-isoxazolepropionic acid receptor
A1R	Adenosine-1 receptor
A2AR	Adenosine A2-receptor
