# Supplementary material for: Non-Competitive AMPA Receptor Antagonist Perampanel Inhibits Ischemia-Induced Neurodegeneration and Behavioral Deficits in Focal Cortical Pial Vessel Disruption Stroke Model
Source: Cells. 2025 Oct 19;14(20):1628. doi: 10.3390/cells14201628 (PMC12562446; doi:10.3390/cells14201628)
Supplement: Supplementary file 1 [file cells-14-01628-s001.zip › cells-3824370-supplementary_Figure S1.pdf]

Supplementary Figure S1:

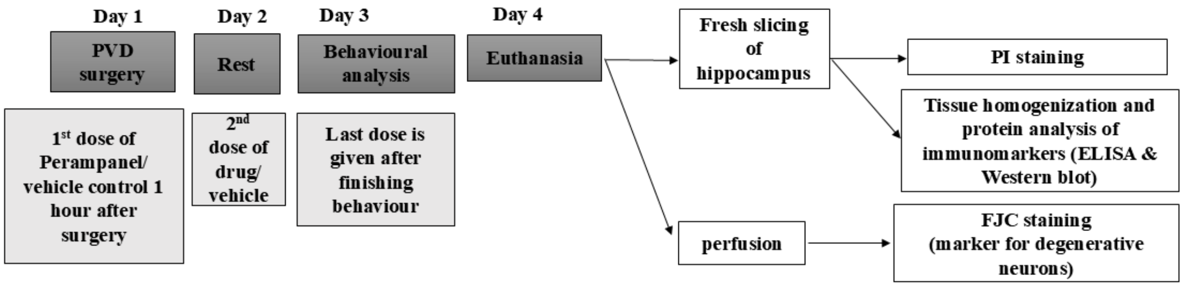

Supplementary Figure S1. Experimental plan involving ischemic stroke induction with pial vessel disruption (PVD) protocol and treatment groups (vehicle control vs. perampanel, given intraperitoneally) to investigate the potential neuroprotective effects of perampanel. PVD is described in detail in the methods section. Rats subjected to PVD received either vehicle control or perampanel 1 hr after PVD procedure. Behavioural tasks were performed on the third day to assess post-stroke memory and other behavioural deficits. On the fourth day, animals were sacrificed for post-mortem analysis (electrophysiology, propidium iodide (PI) and FluoroJade-C (FJC) staining, and biochemical analysis).
