# Supplementary material for: Non-Competitive AMPA Receptor Antagonist Perampanel Inhibits Ischemia-Induced Neurodegeneration and Behavioral Deficits in Focal Cortical Pial Vessel Disruption Stroke Model
Source: Cells. 2025 Oct 19;14(20):1628. doi: 10.3390/cells14201628 (PMC12562446; doi:10.3390/cells14201628)

Supplementary Figure S2. Representative images for Figure 8 (propidium iodide) and Figure 9 (FluoroJade-C).

Representative Images For figure 8

PVD-Ipsi

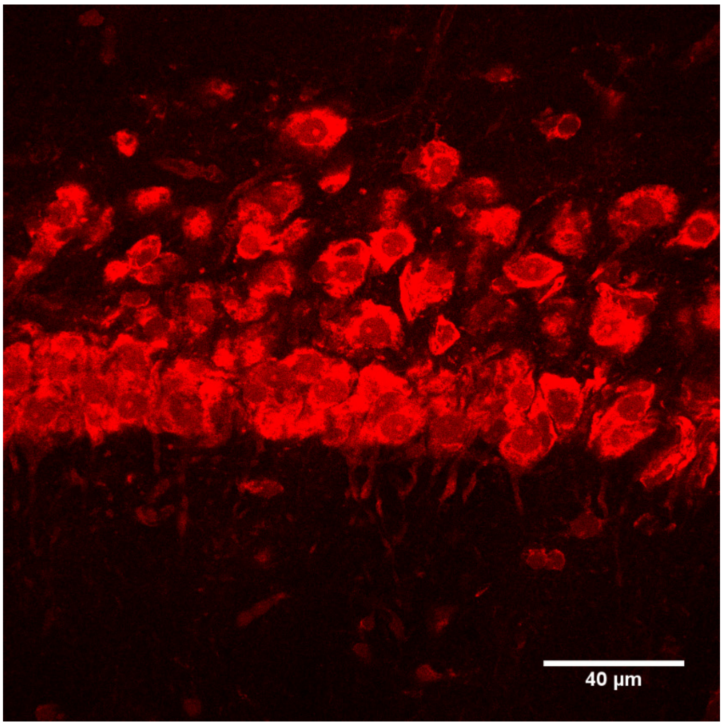

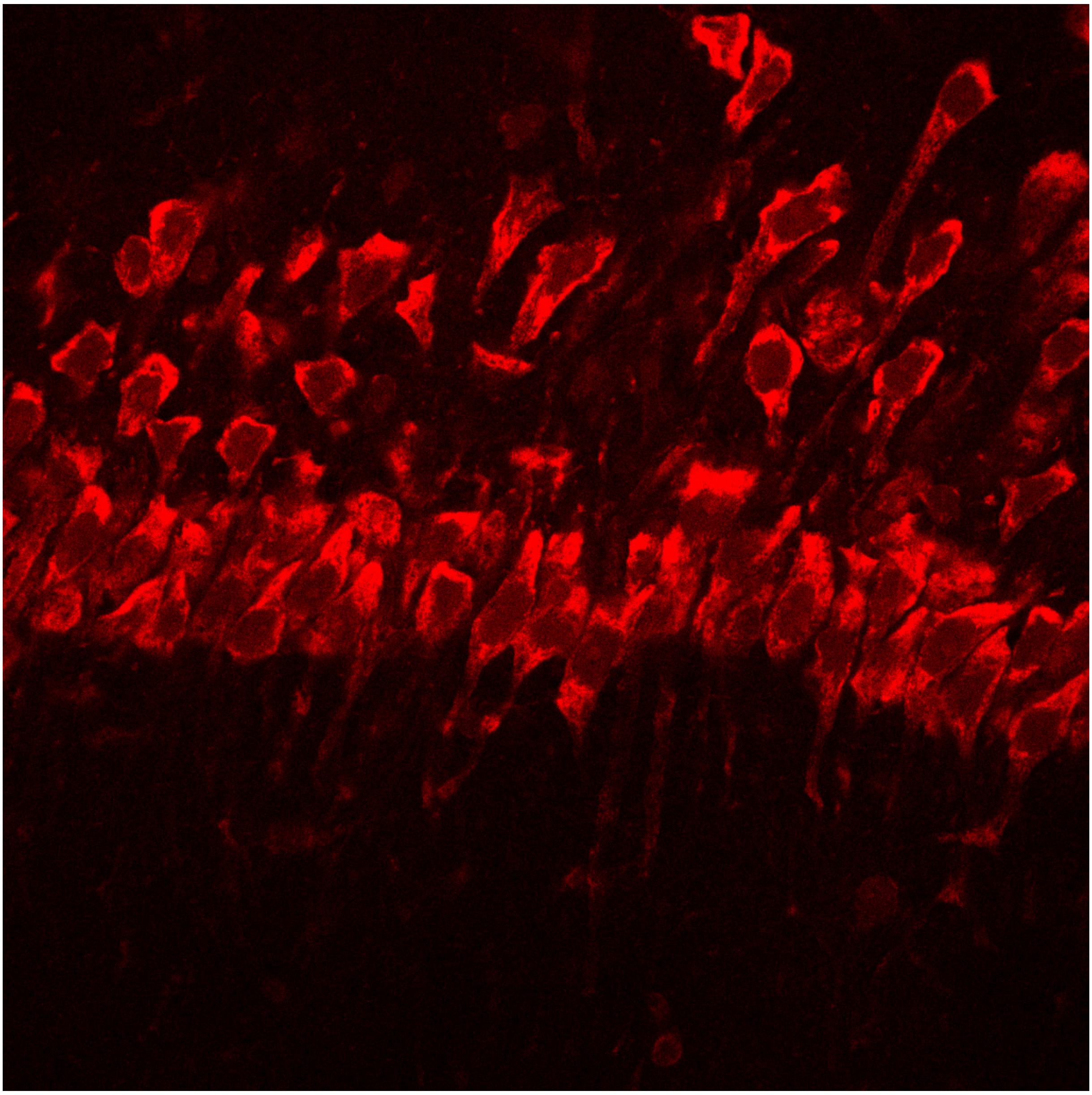

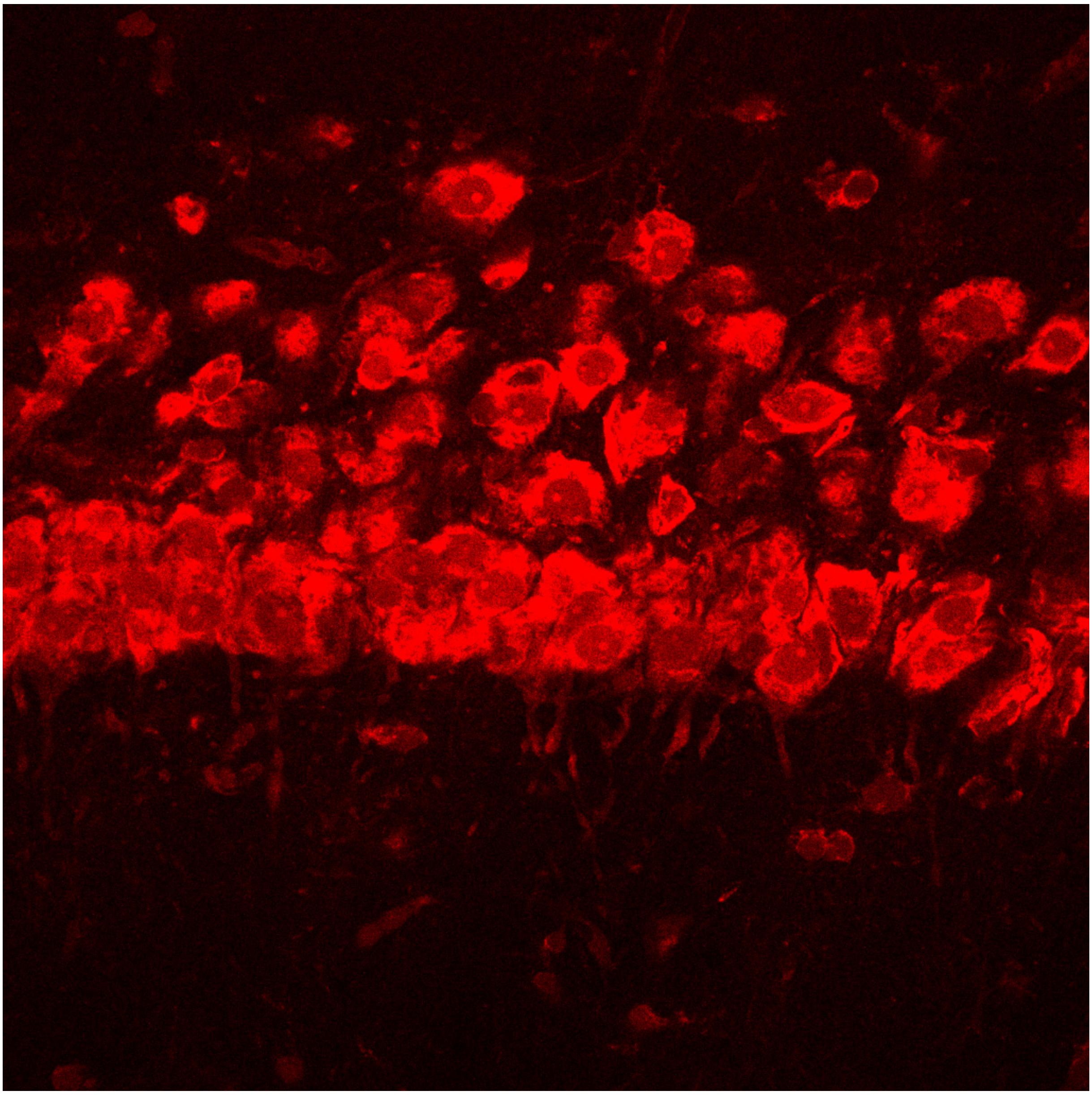

PVD-Contralateral

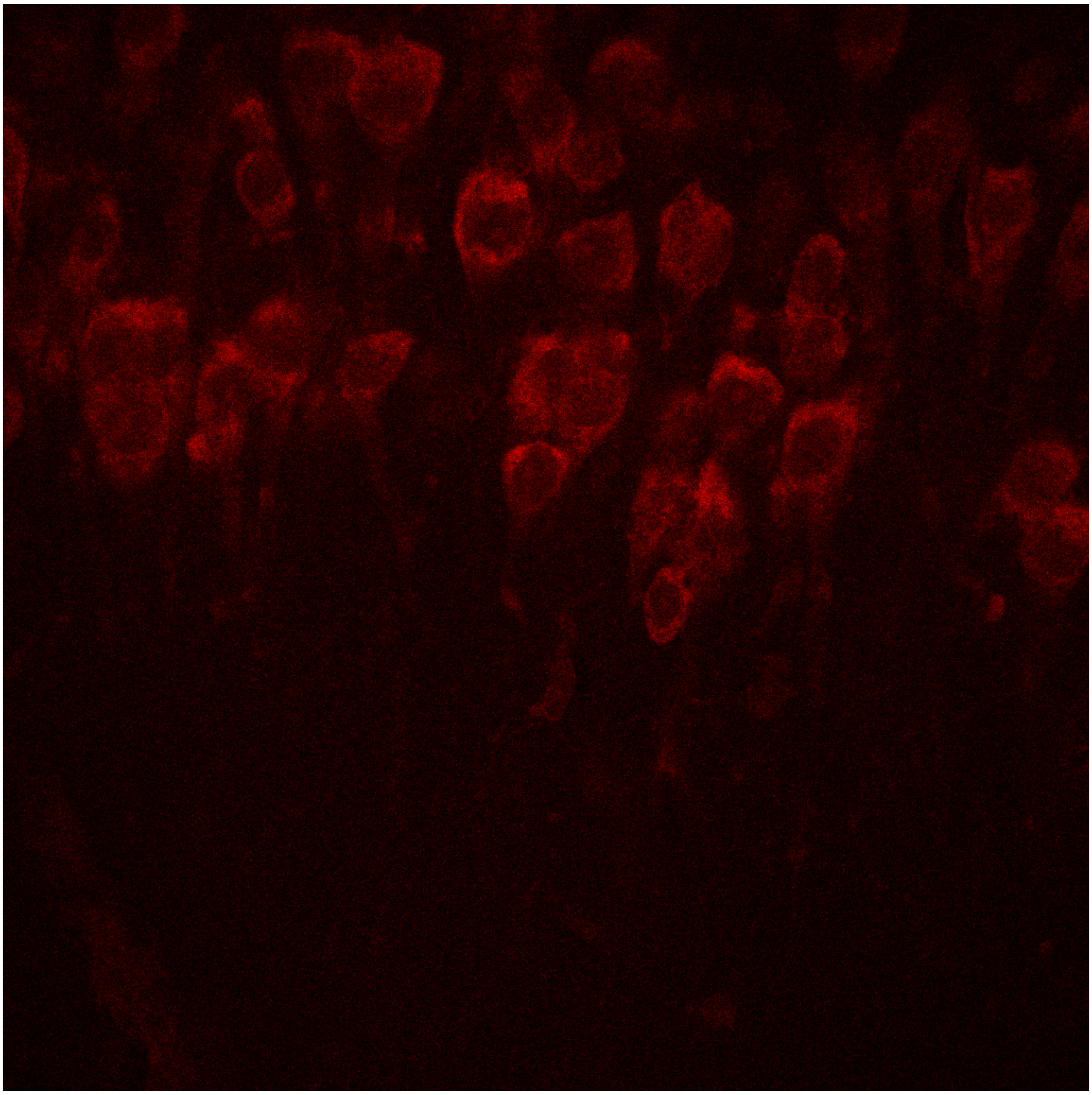

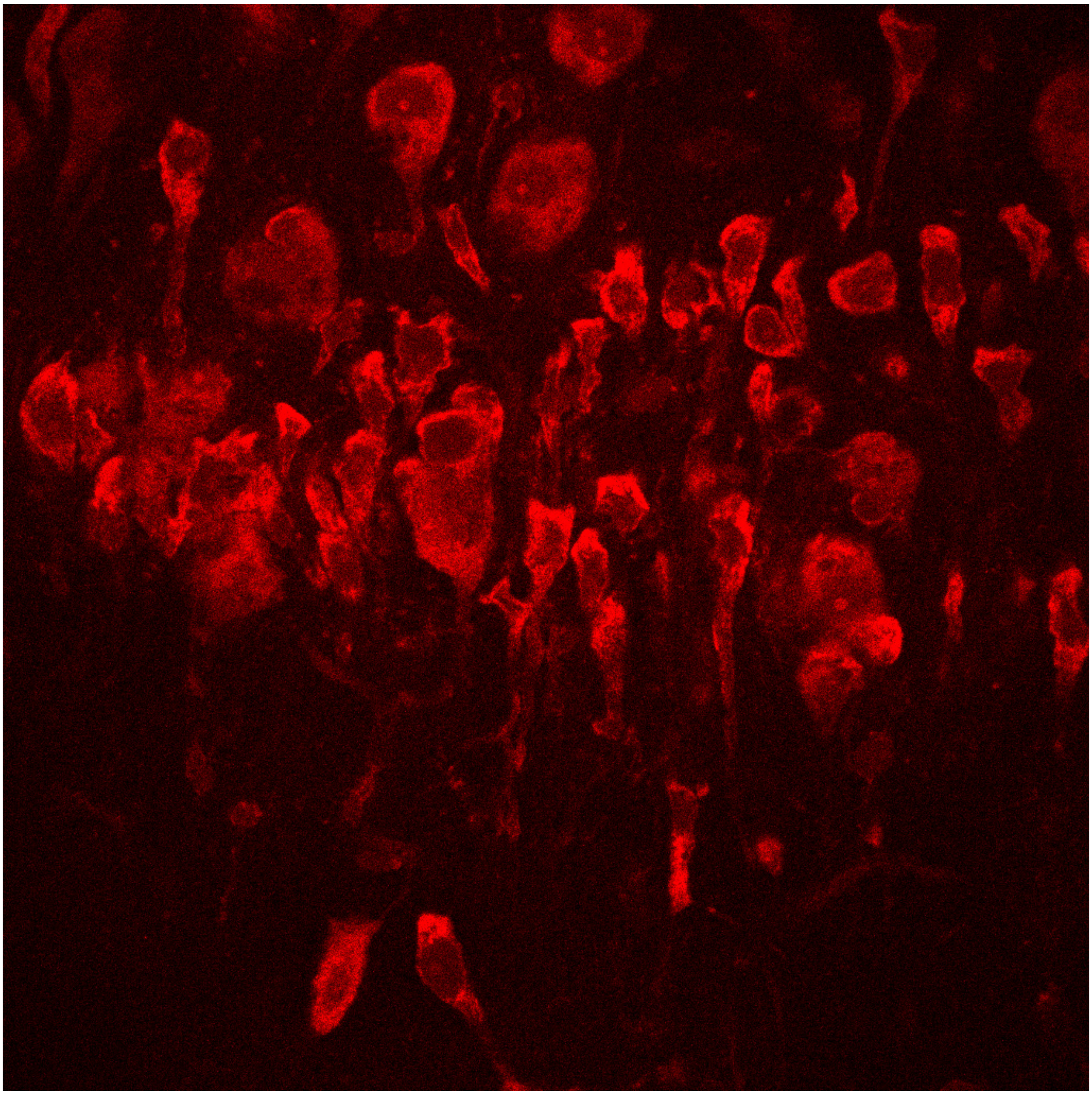

PVD/Perampanel-Ipsi

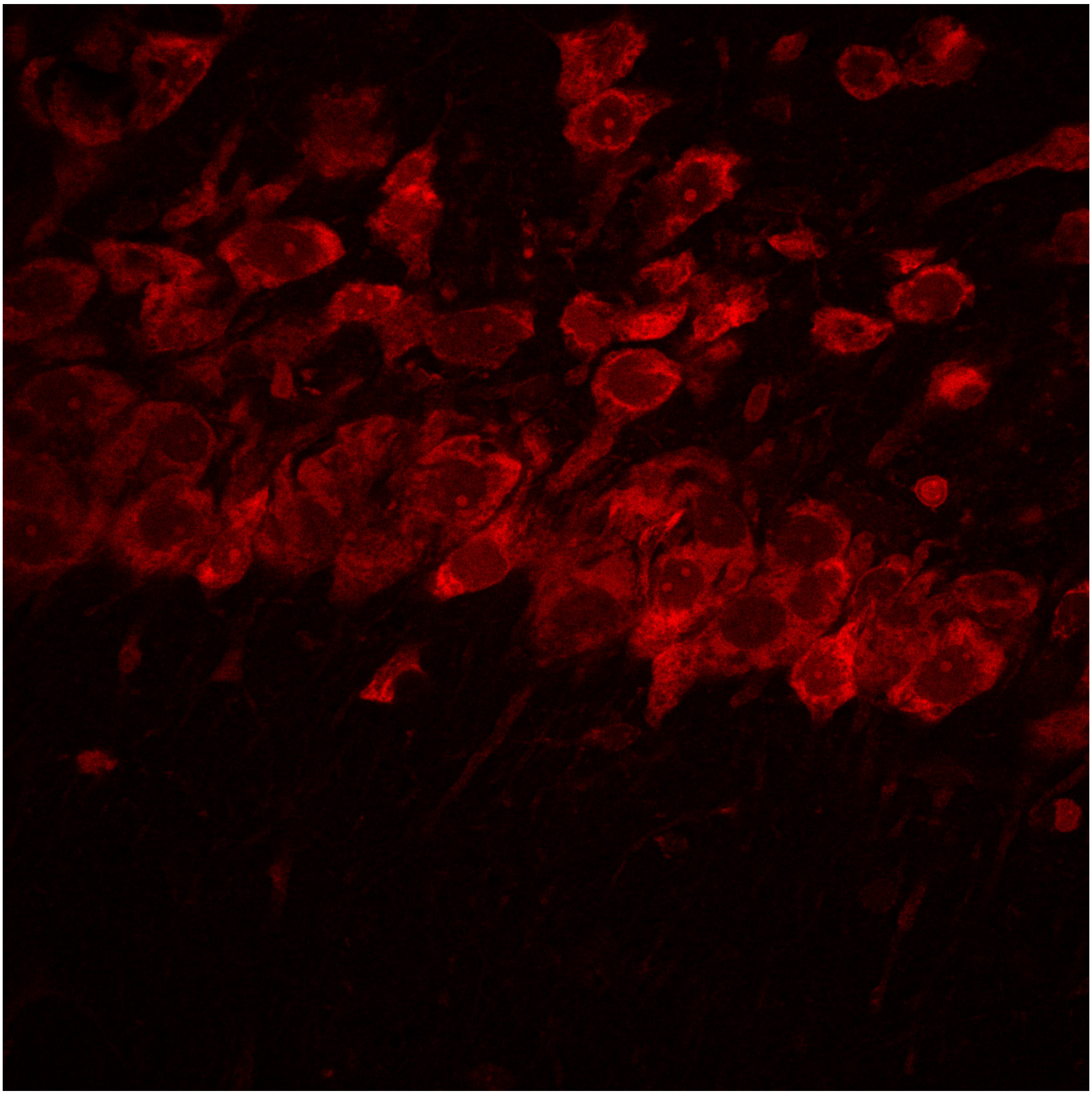

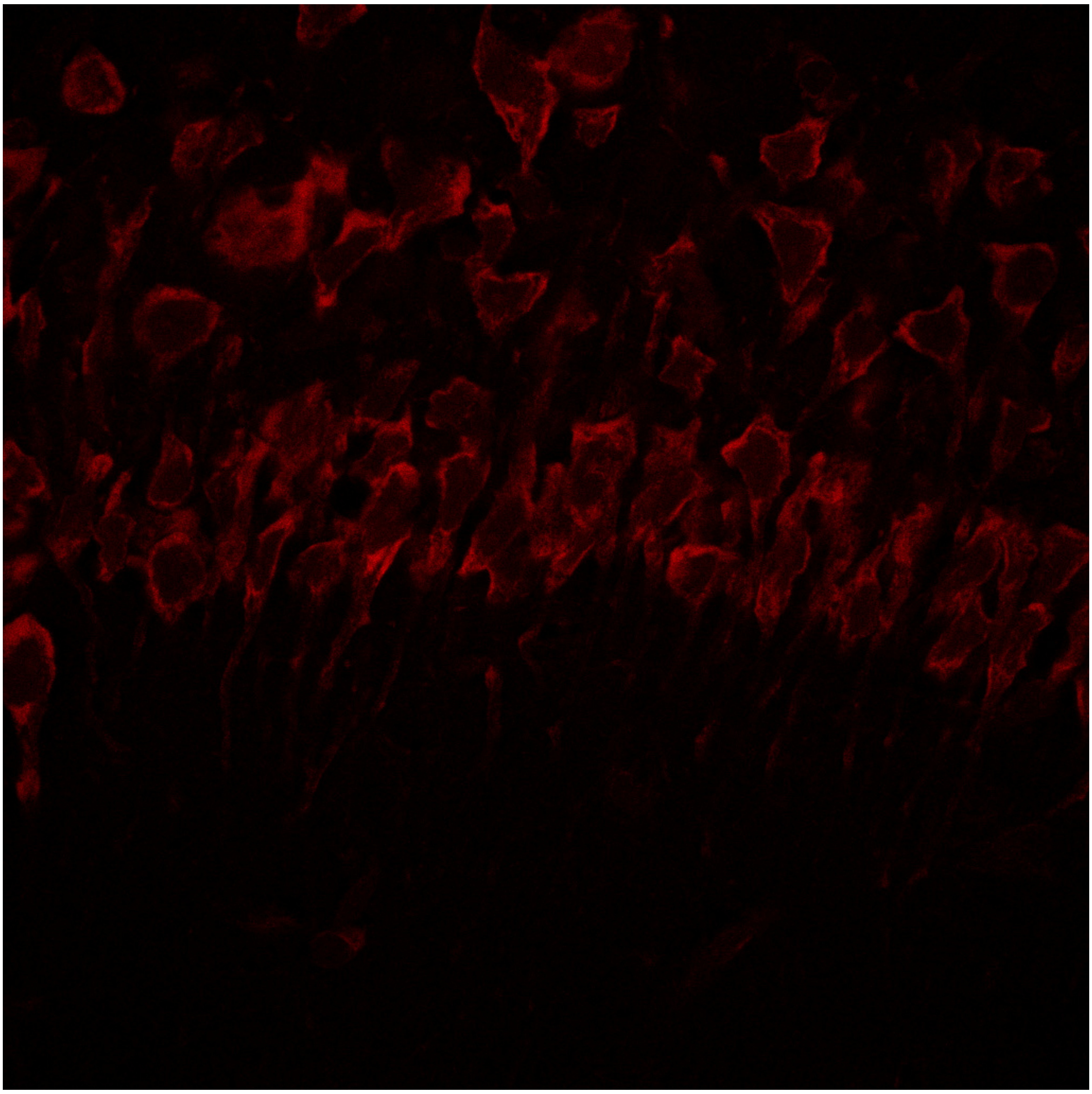

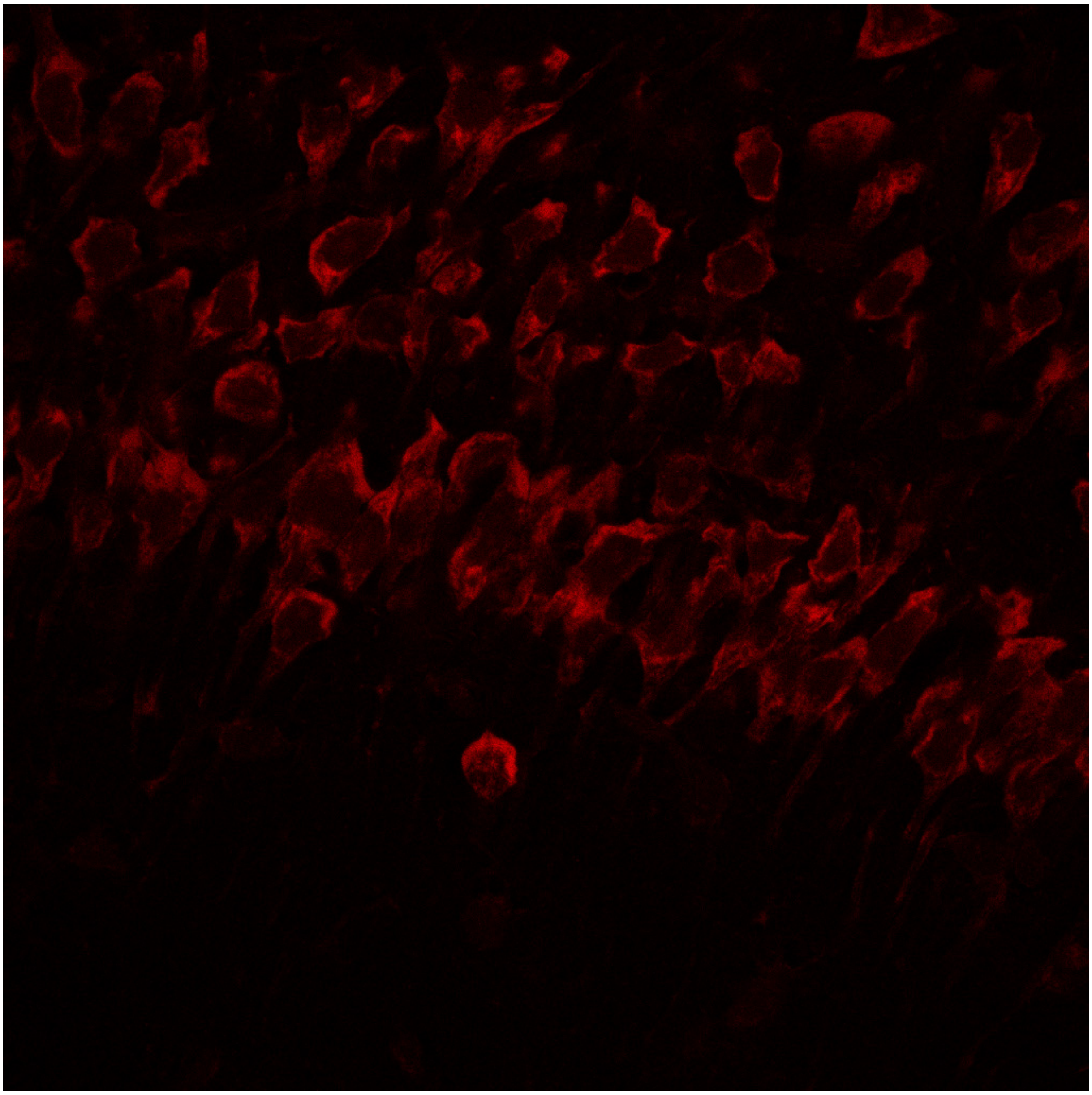

PVD/Perampanel-Contra

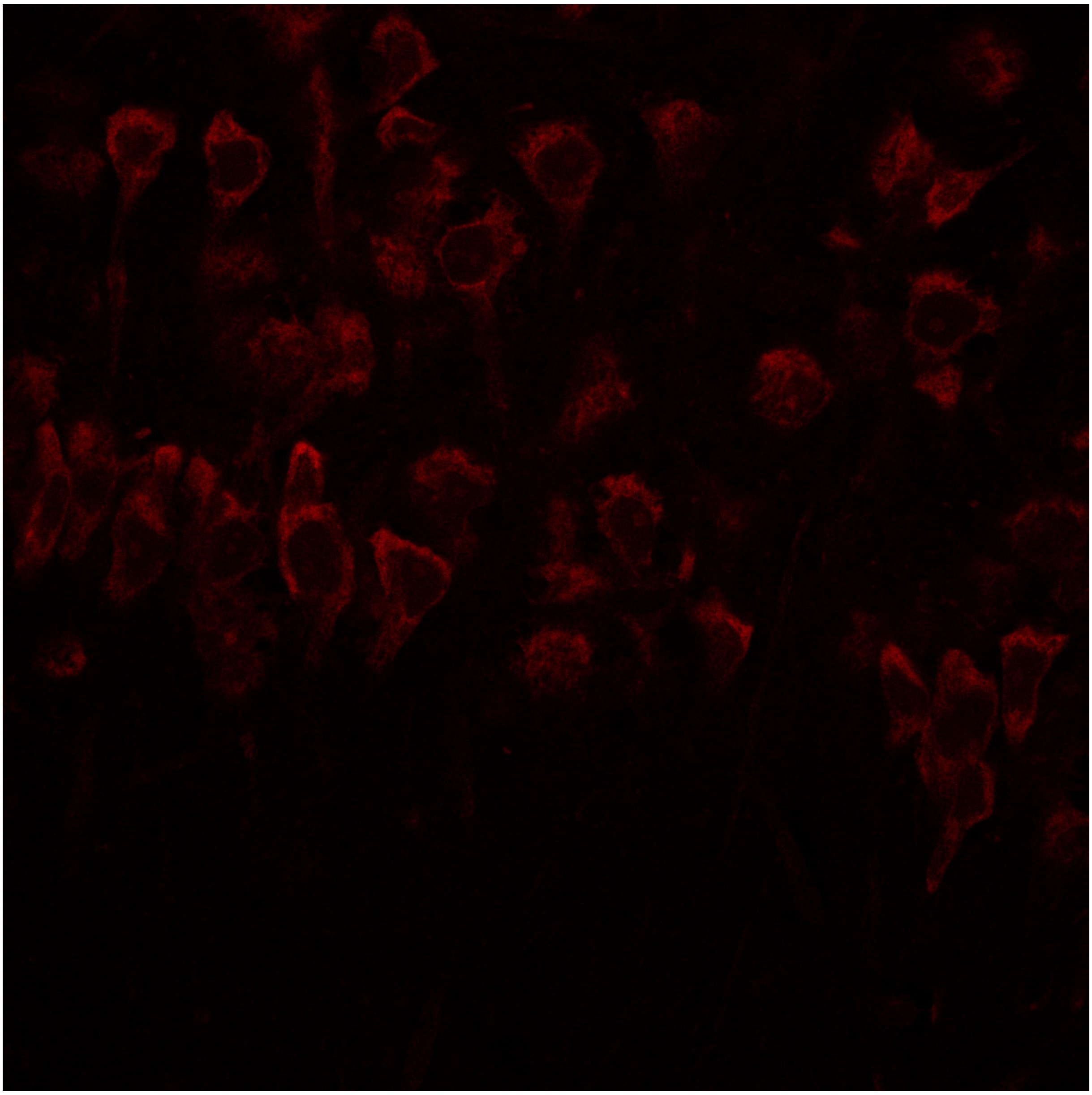

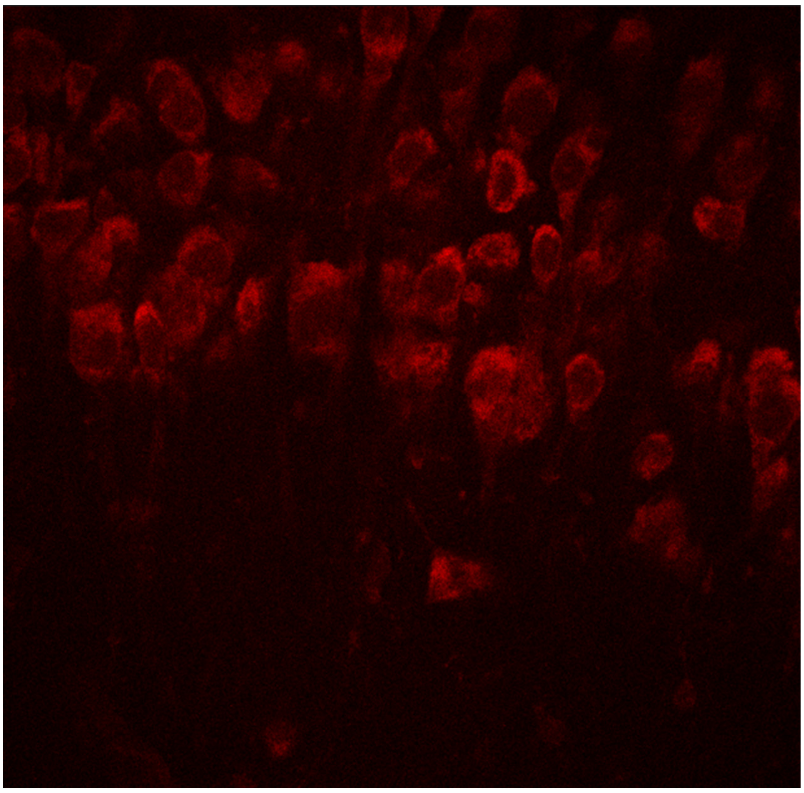

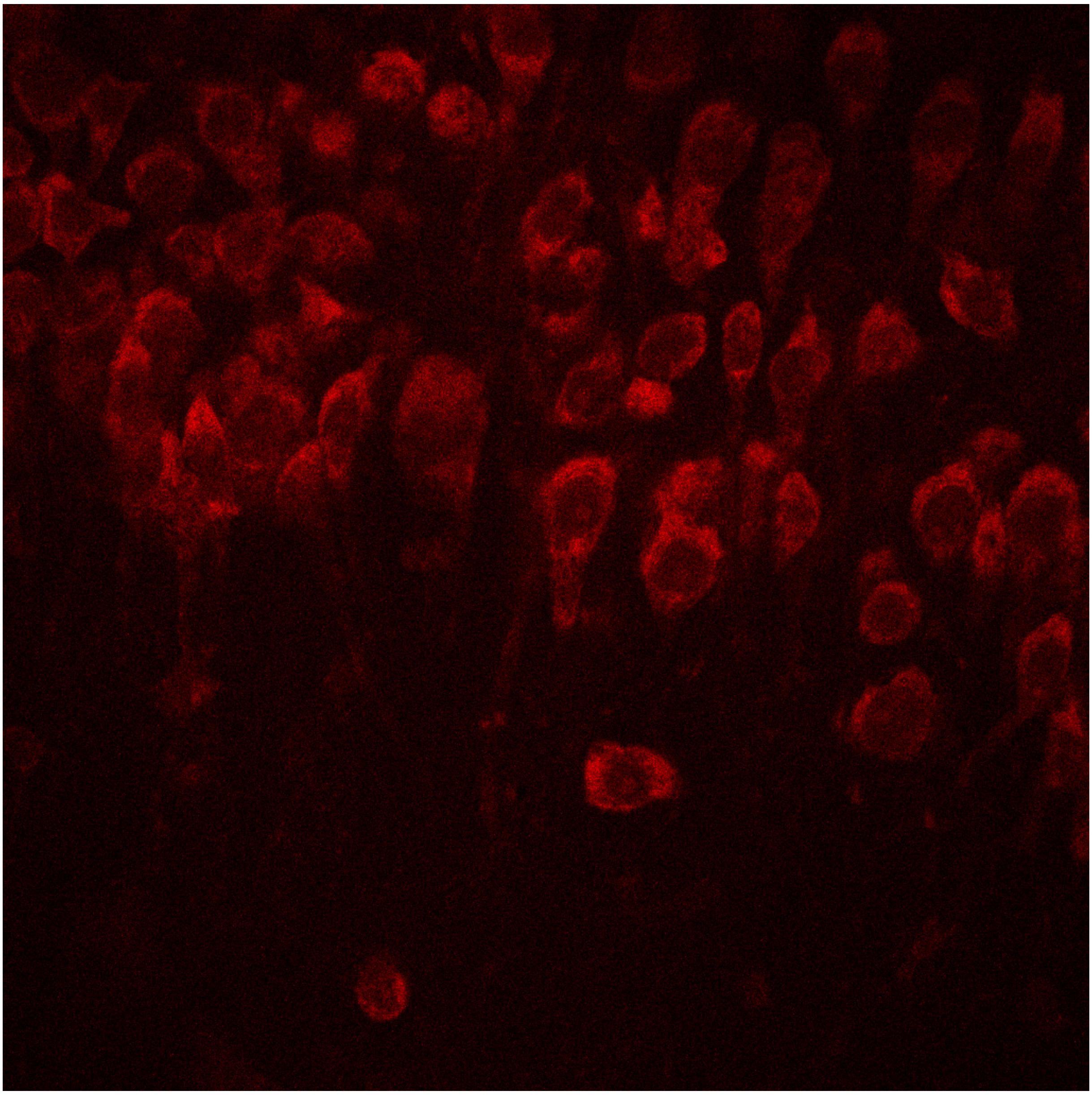

SHAM-Ipsi

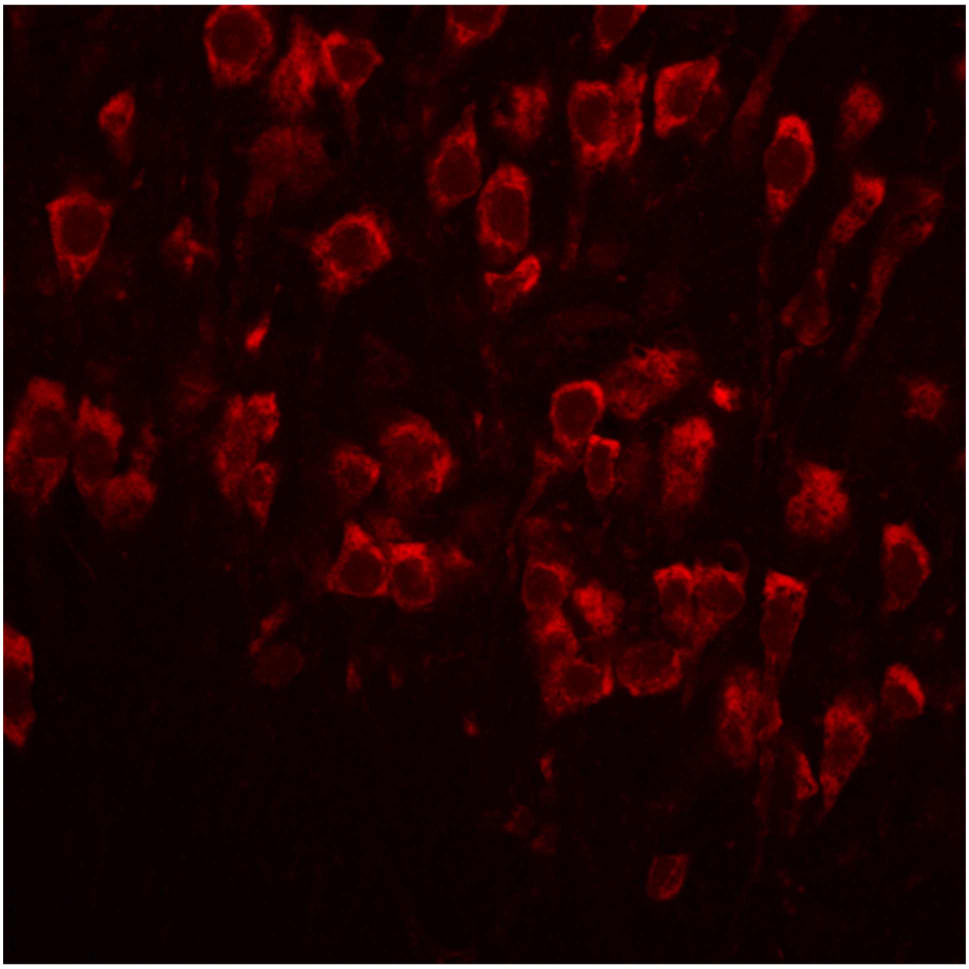

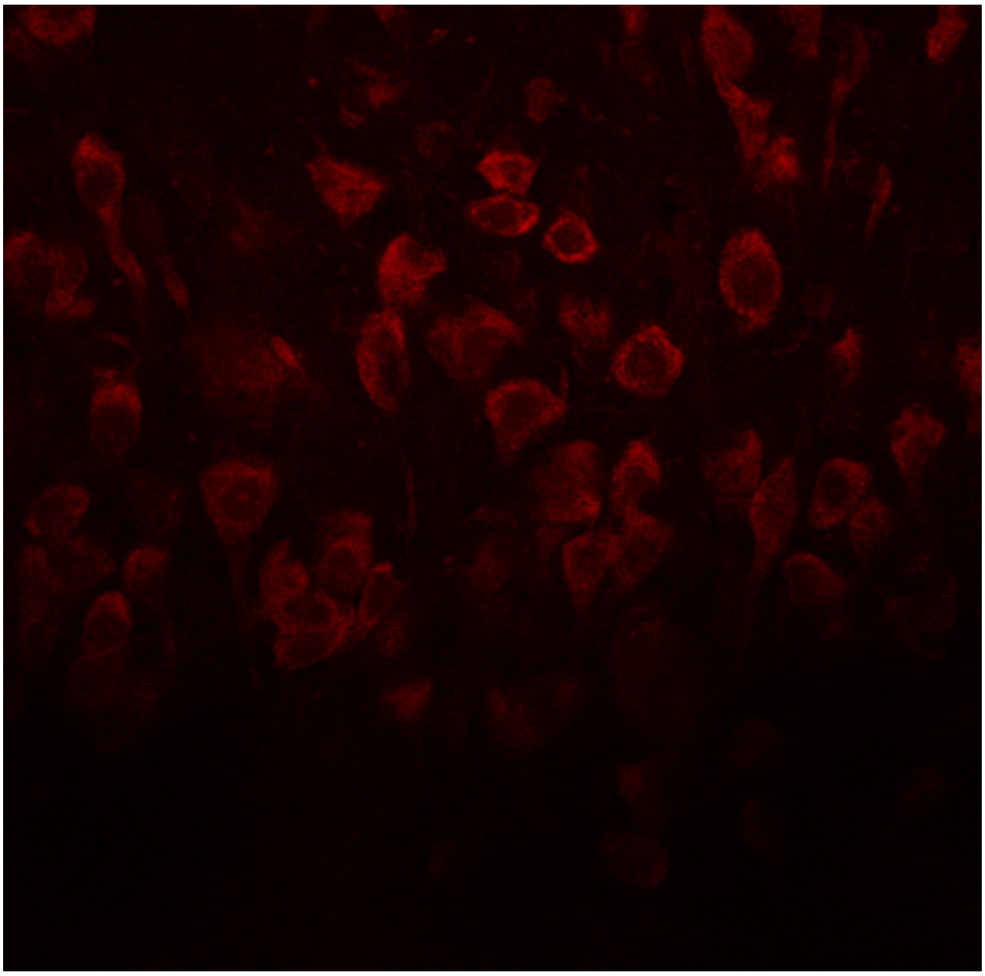

Sham-Contra

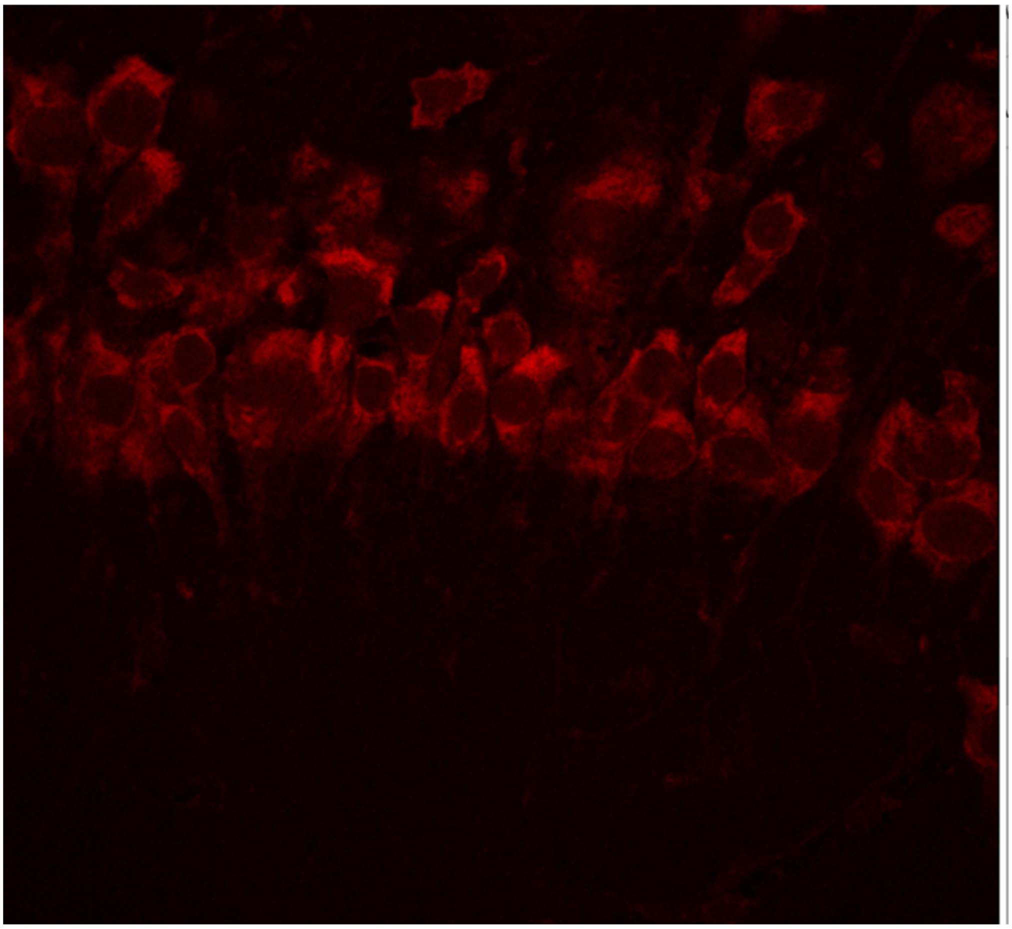

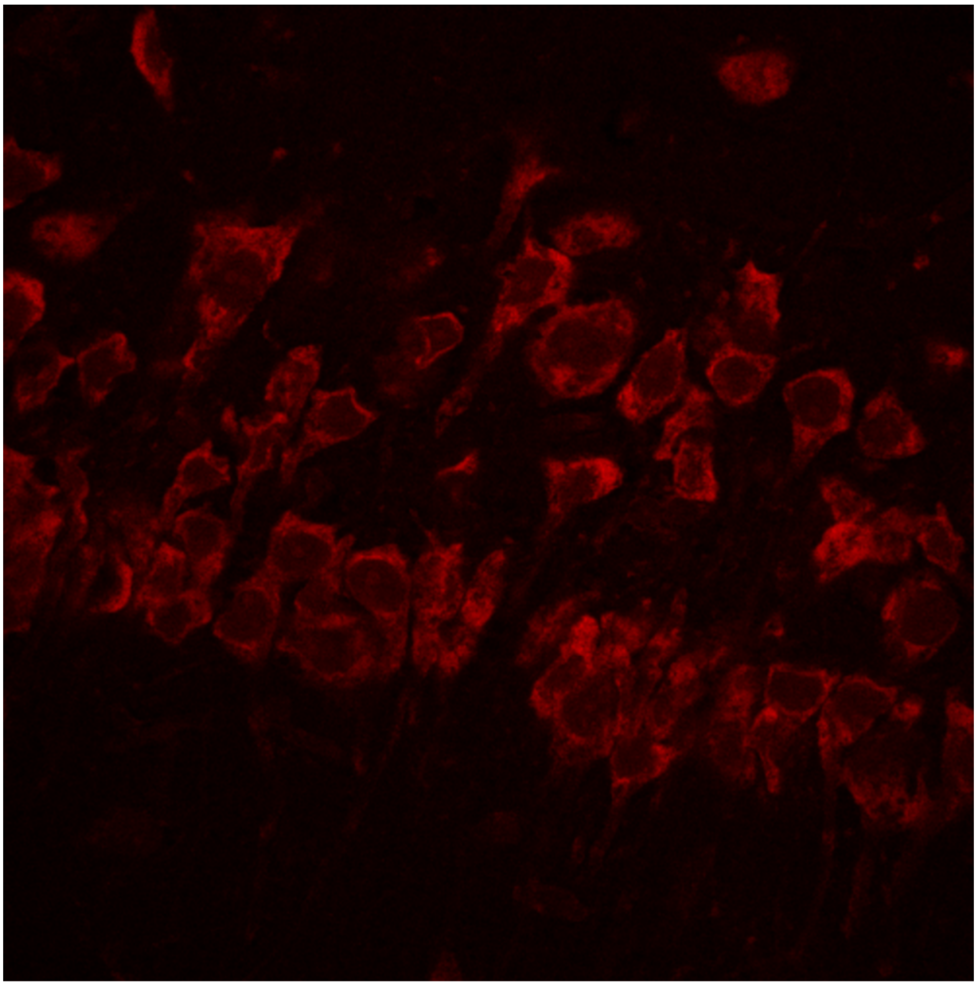

Representative Images Figure9: FJC

PVD-Perampanel Ipsi

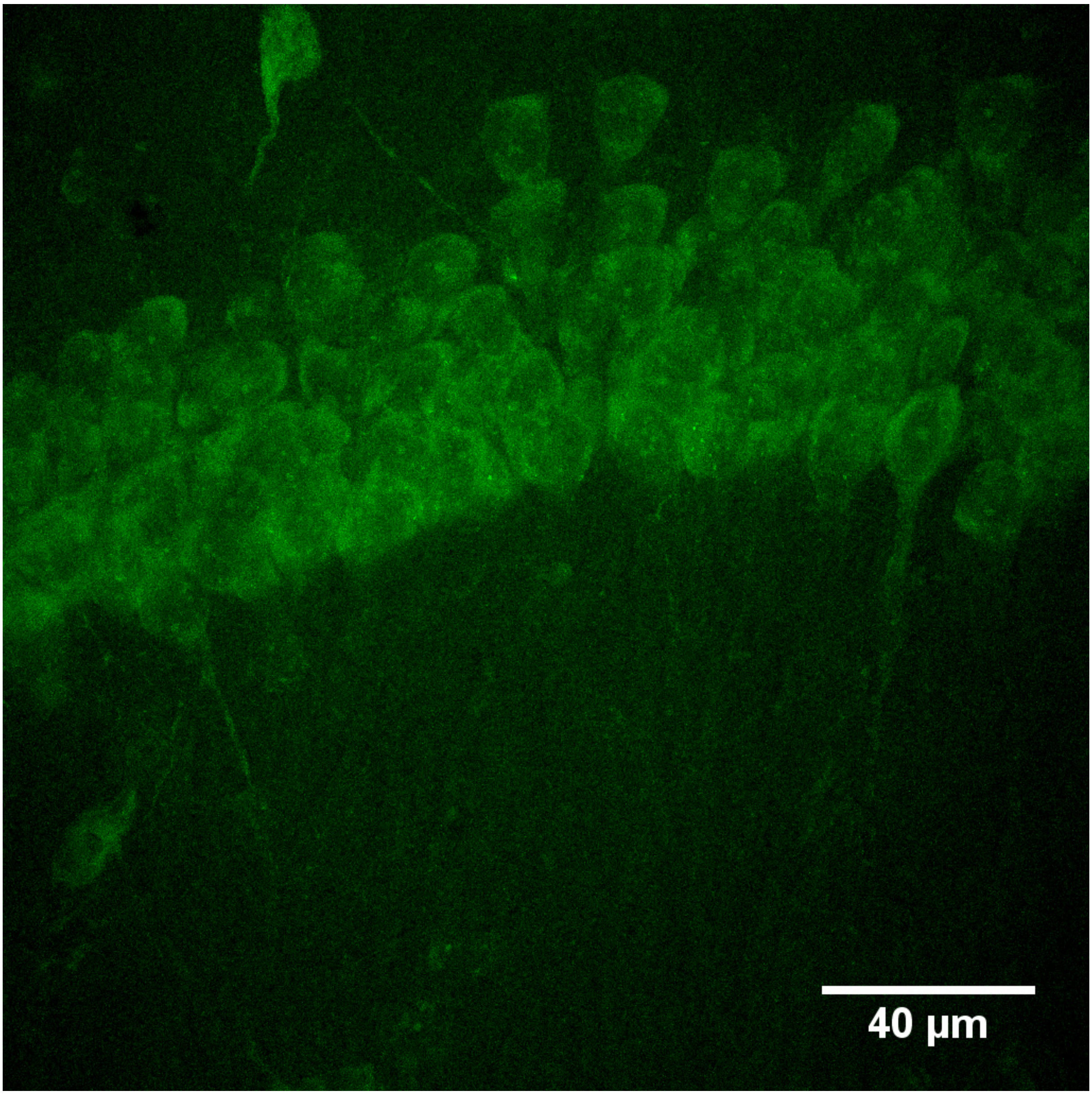

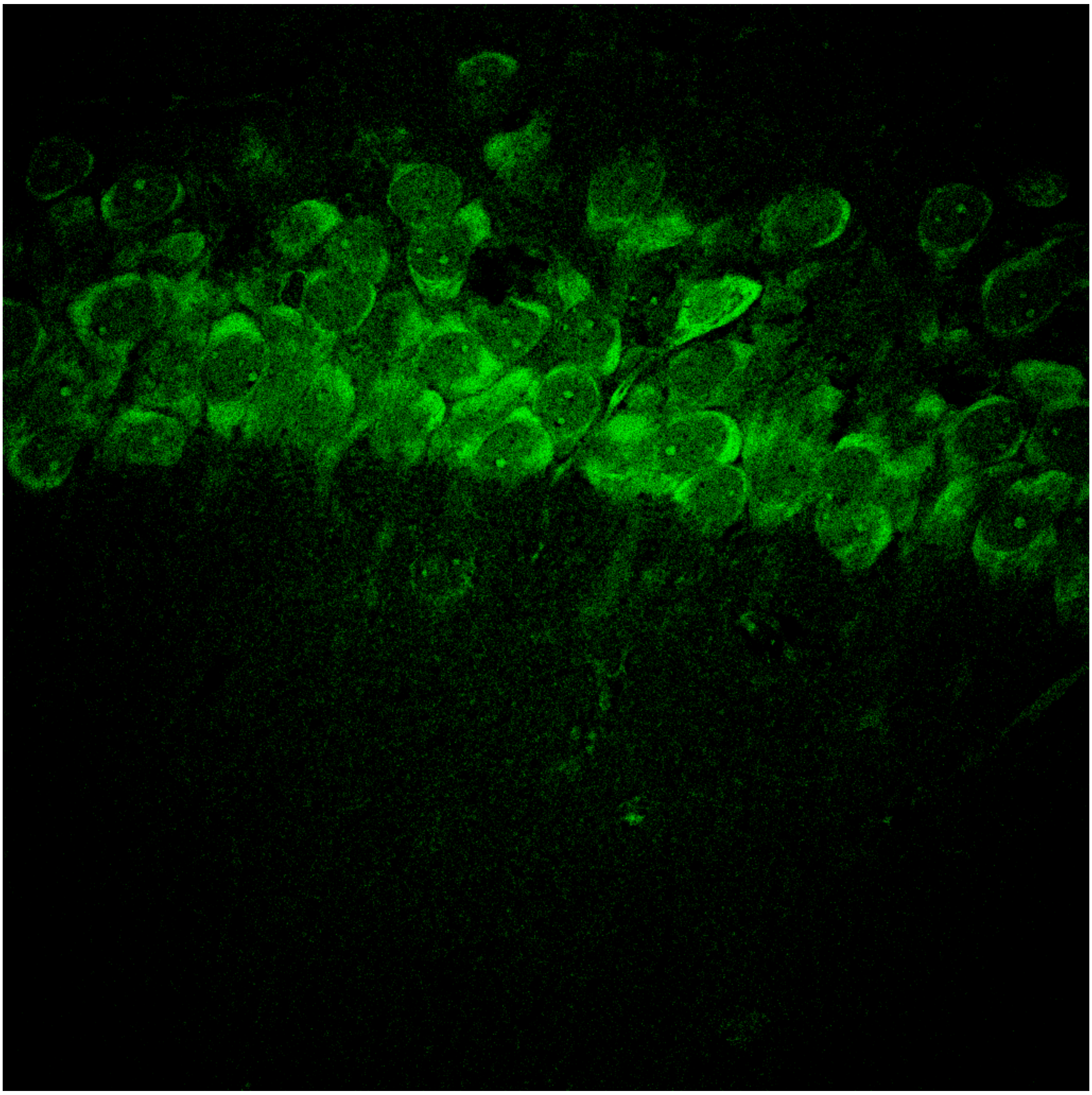

PVD-Perampanel Contra

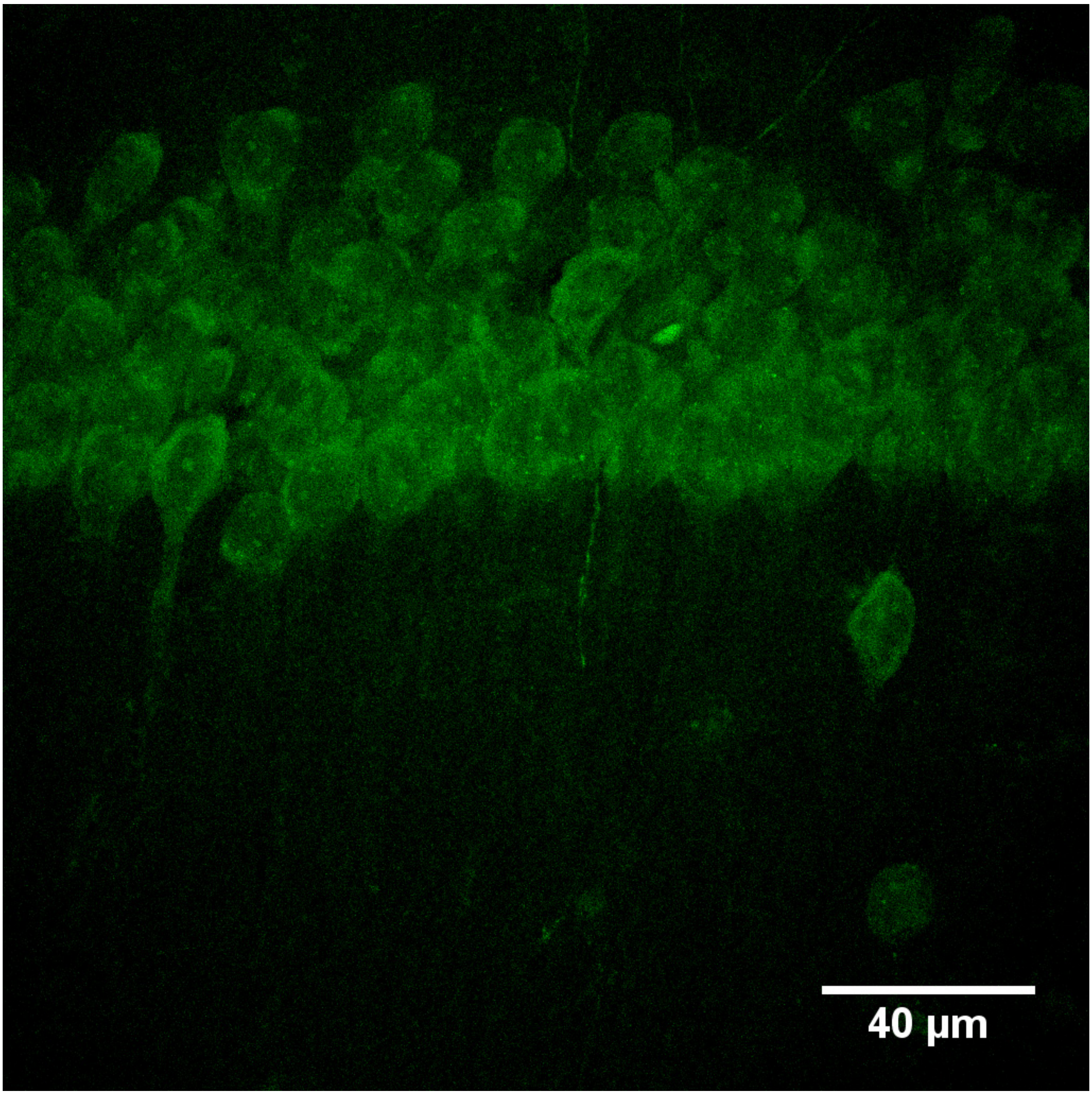

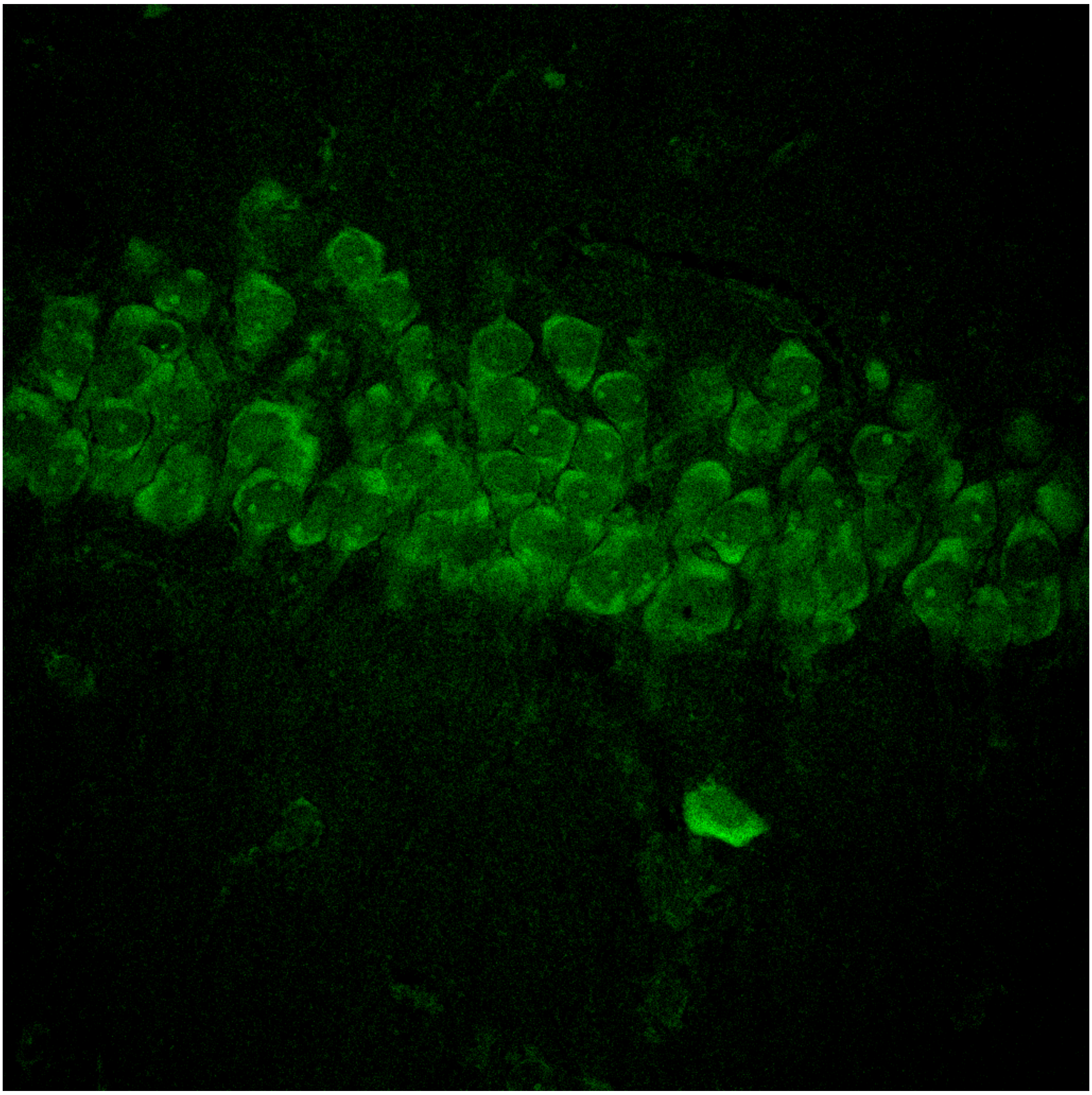

PVD-Ipsi

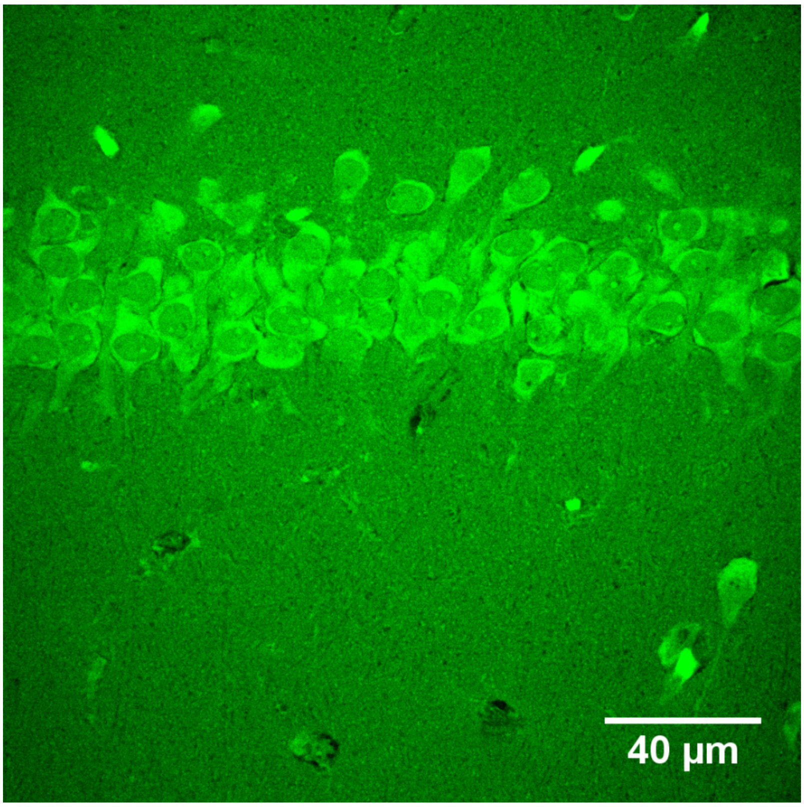

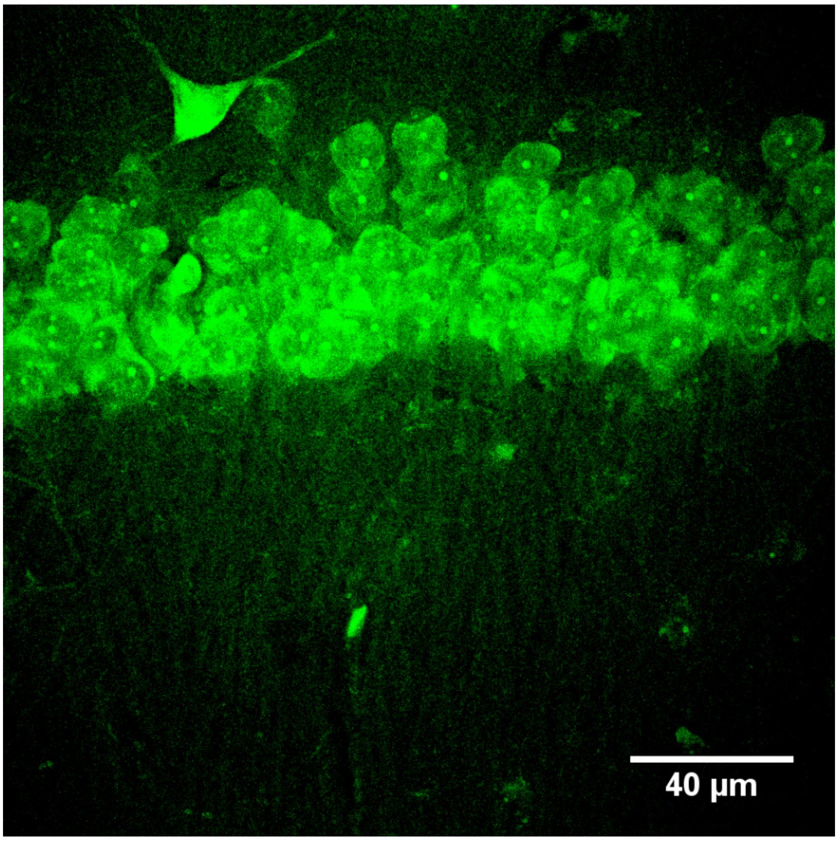
40 µm

PVD-Contra

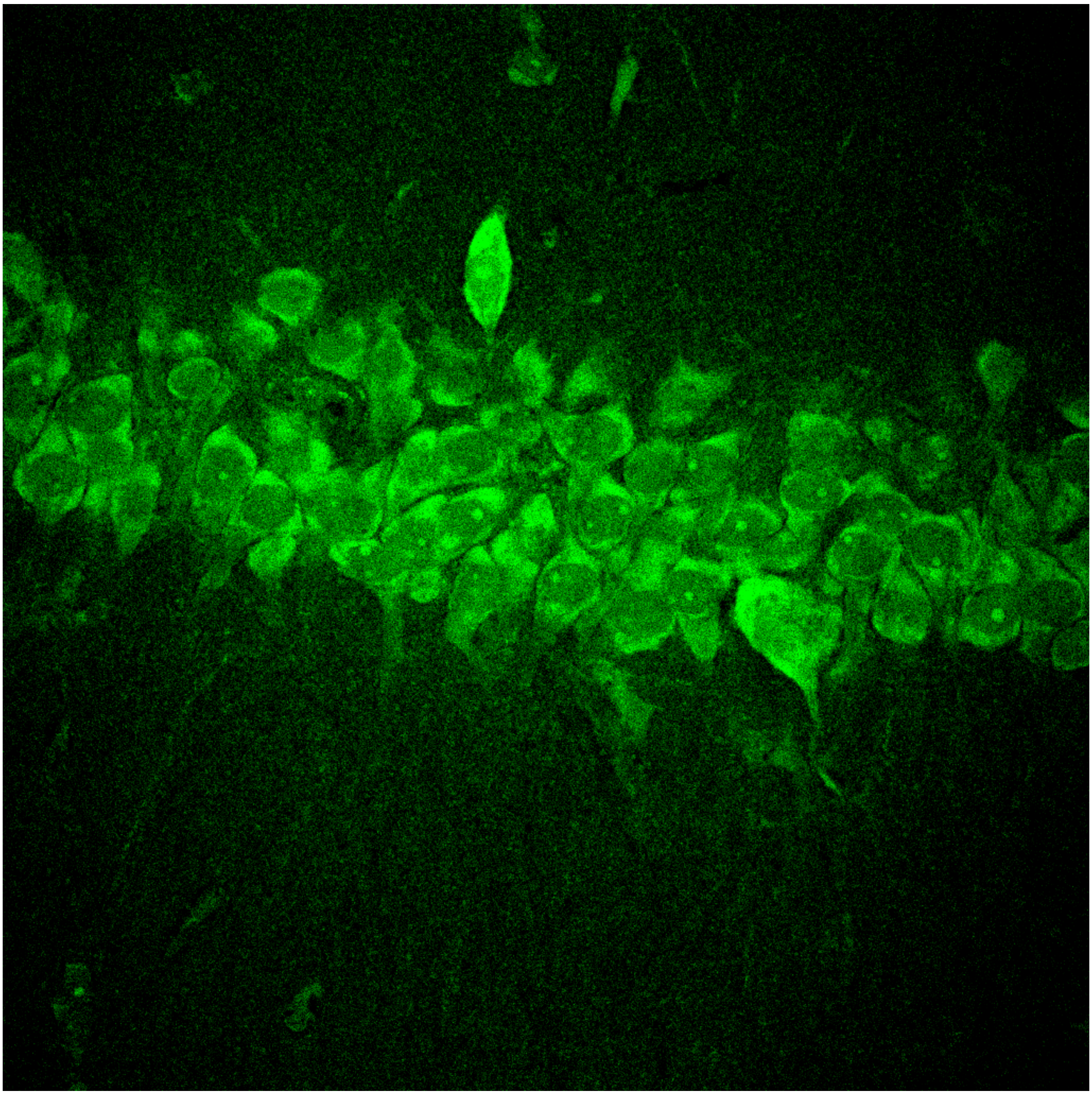

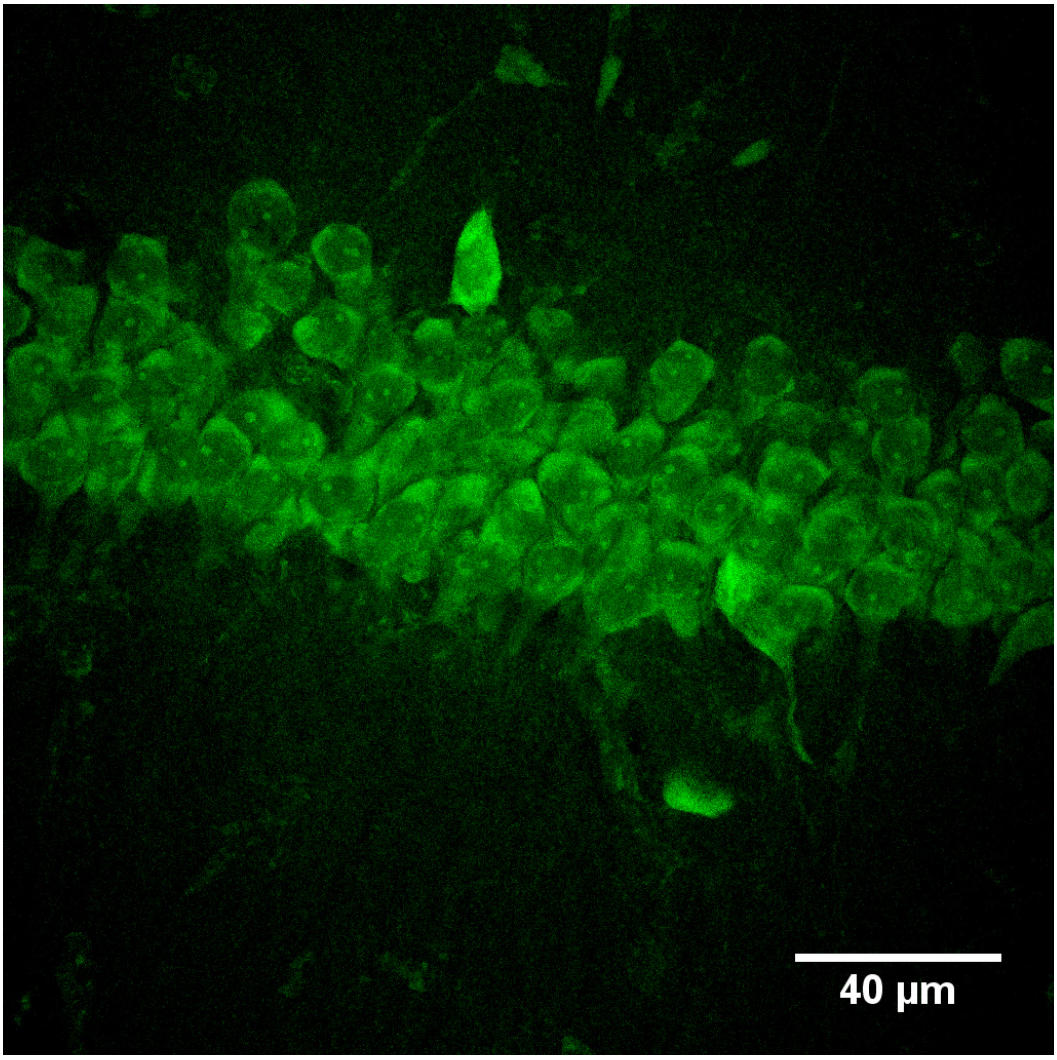
40 µm

SHAM-Ipsi

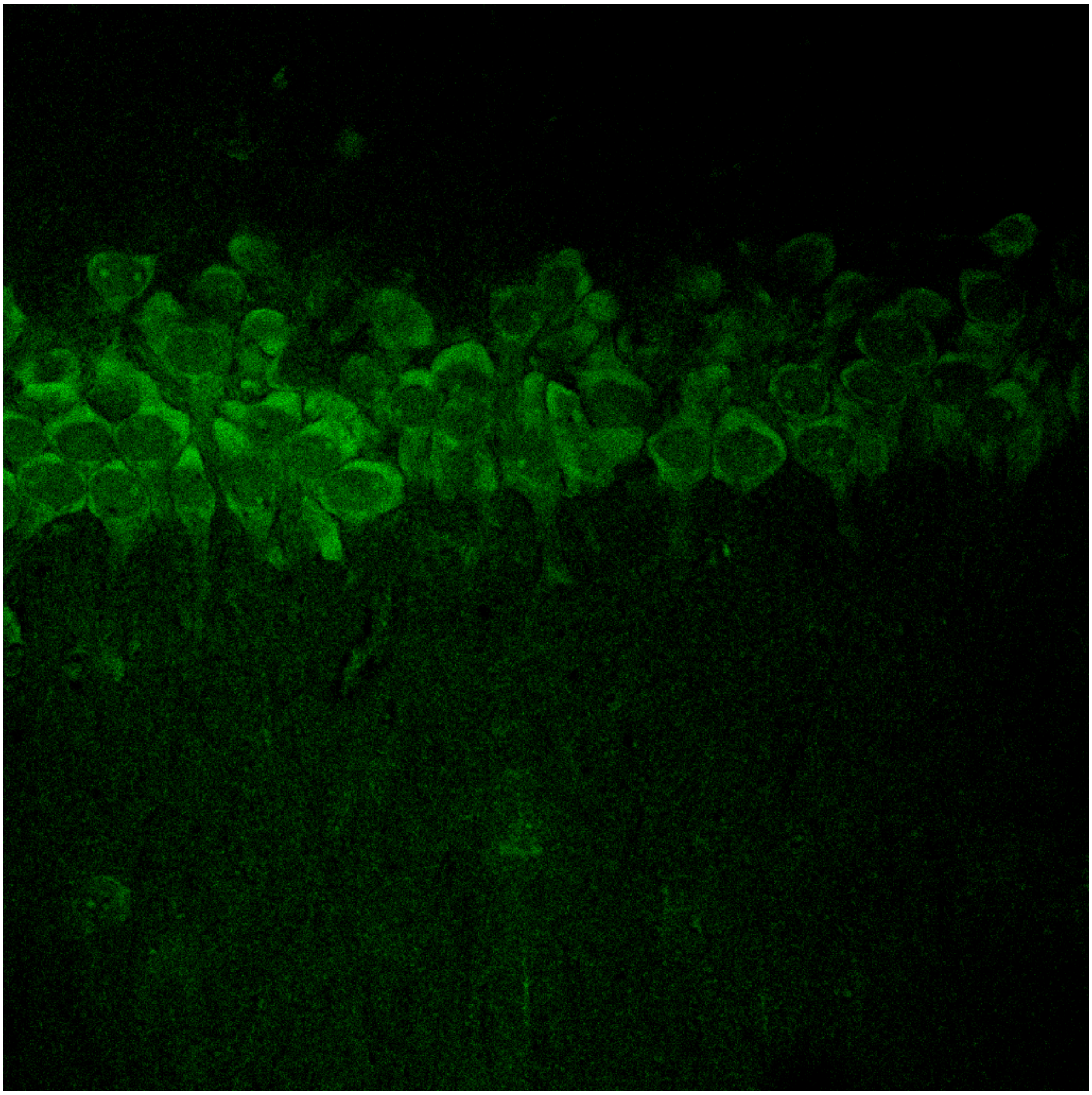

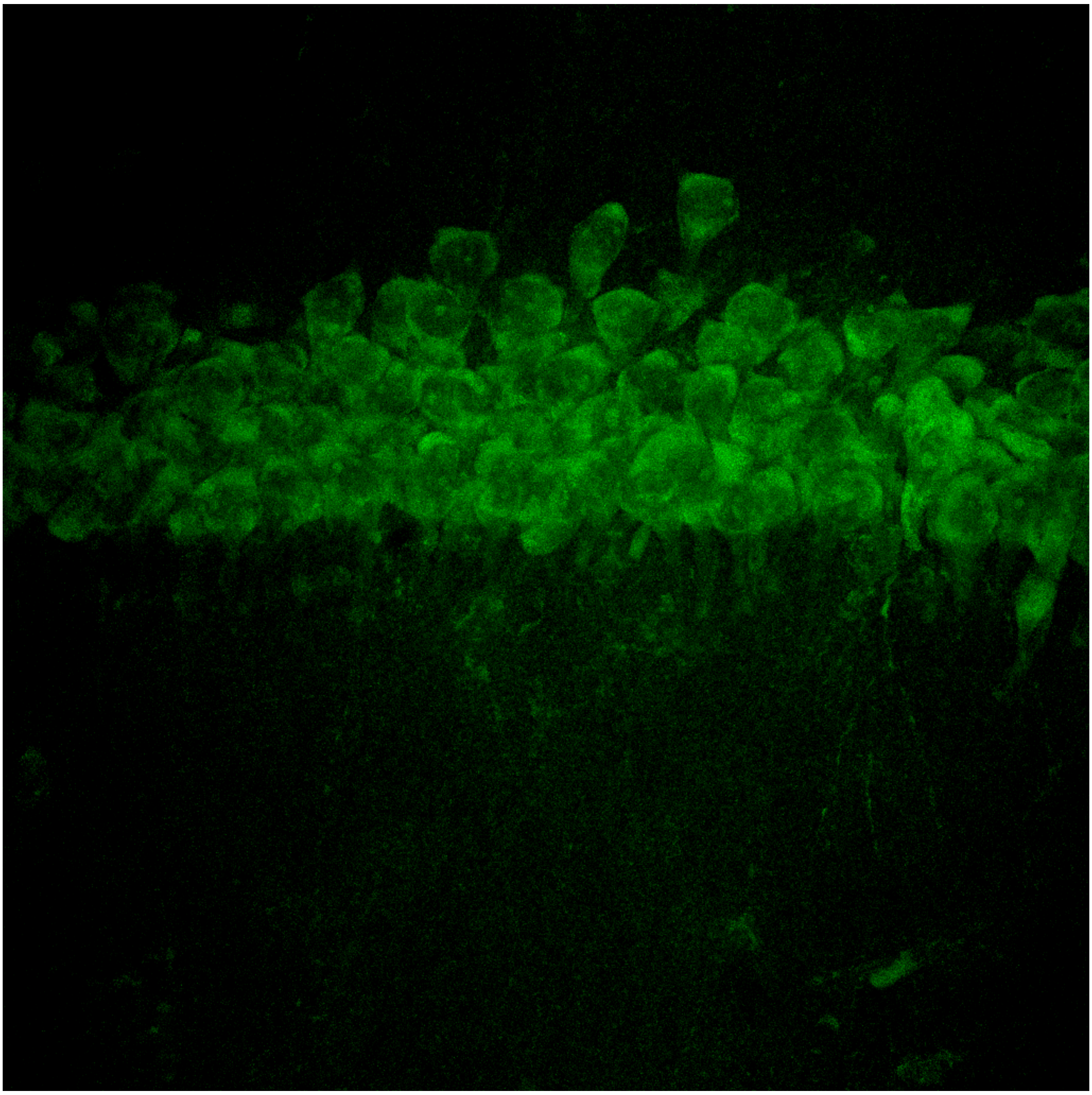

Sham-Contra

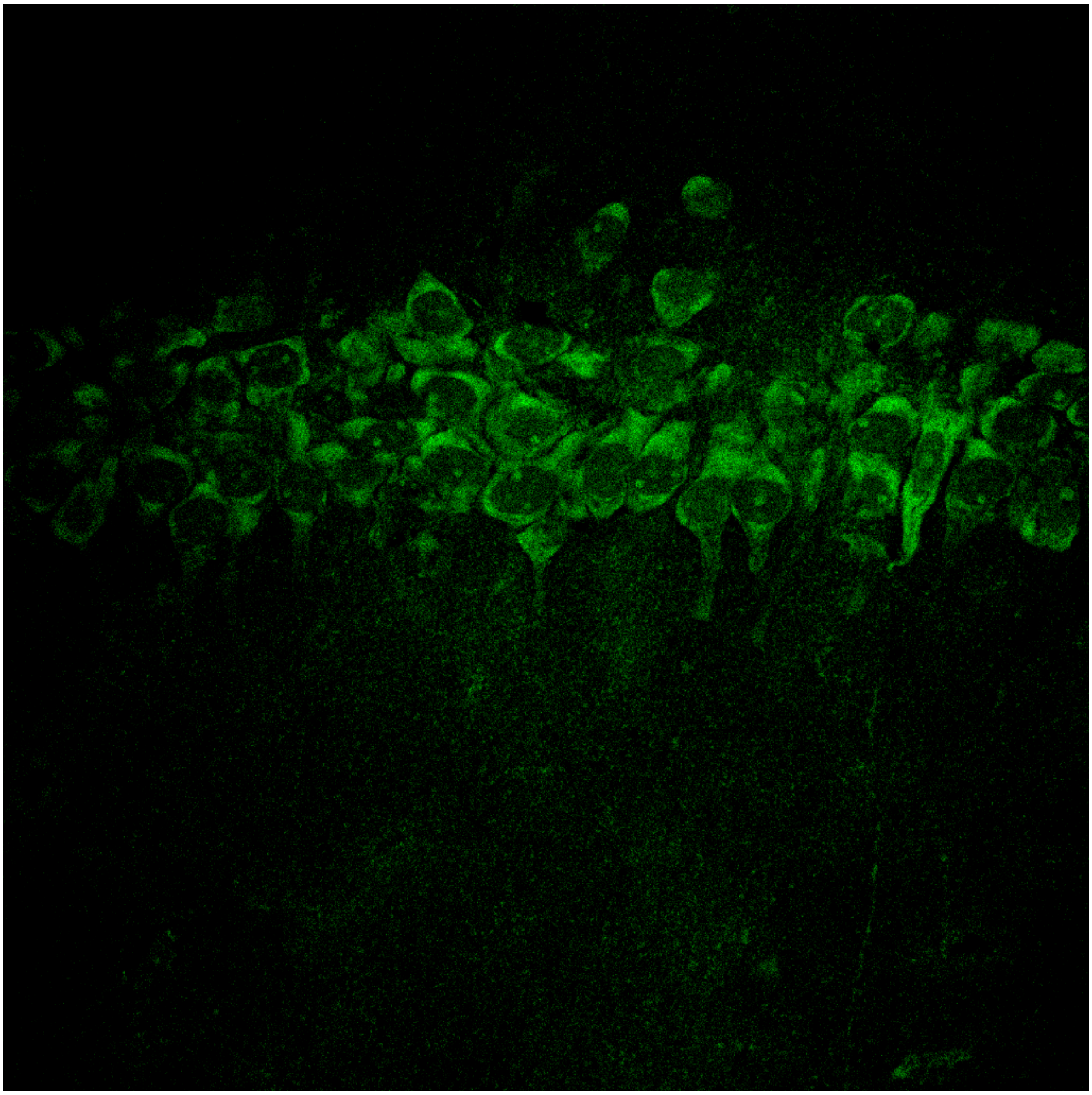

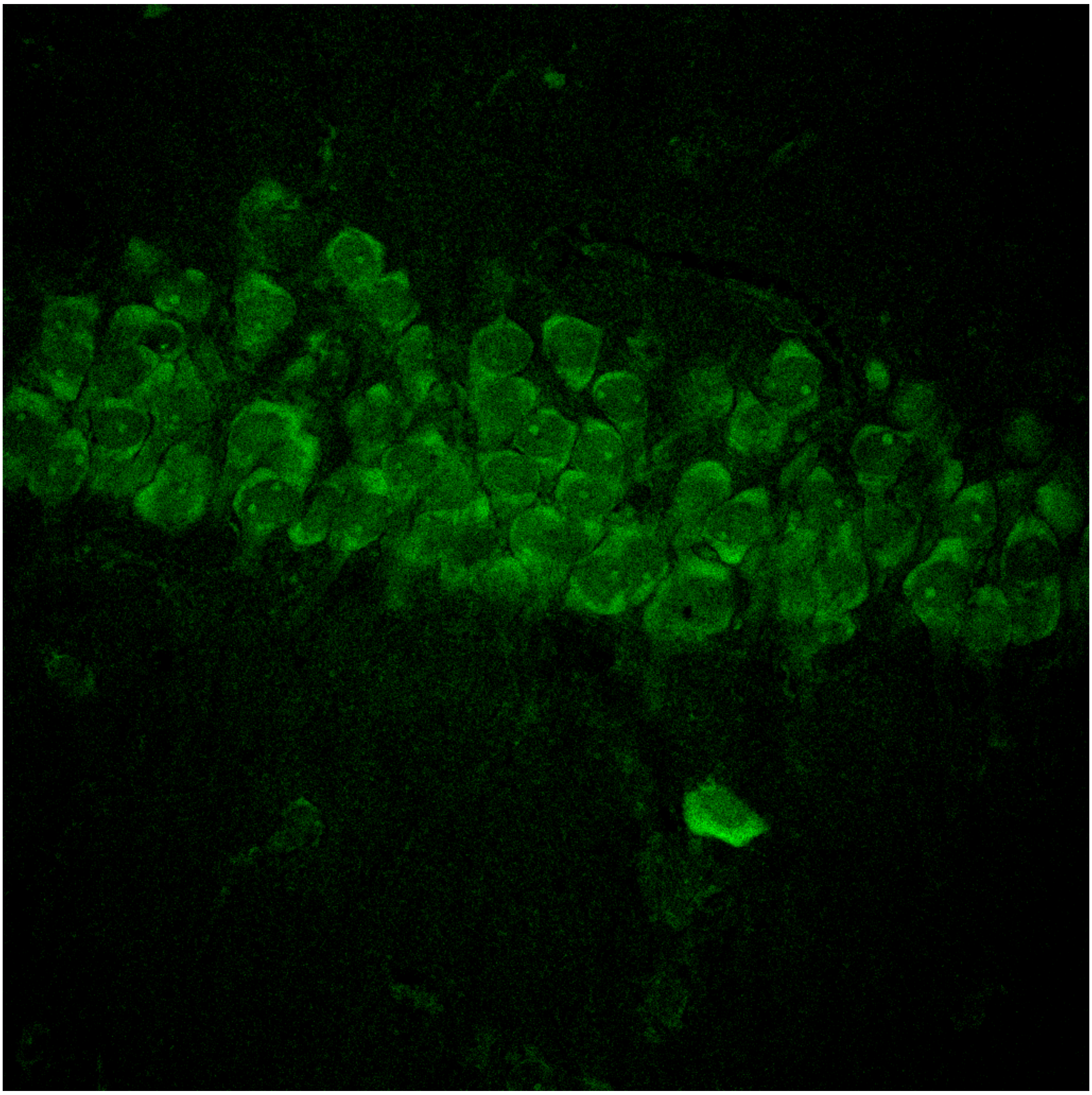

Supplement: Supplementary file 1 [file cells-14-01628-s001.zip › Supplementary Figure S2_Representative images for Figures 8 and 9.pdf]
