# Supplementary material for: Non-Competitive AMPA Receptor Antagonist Perampanel Inhibits Ischemia-Induced Neurodegeneration and Behavioral Deficits in Focal Cortical Pial Vessel Disruption Stroke Model
Source: Cells. 2025 Oct 19;14(20):1628. doi: 10.3390/cells14201628 (PMC12562446; doi:10.3390/cells14201628)

Supplementary Figure S3. Pilot study showing changes in NeuN (red), nNOS (green), and DAPI (blue) staining in rats subjected to PVD, Sham control, and PVD + Perampanel. PVD increased nNOS but reduced NeuN compared to either Sham or PVD + Perampanel groups.

PVD (DAPI-Blue, Green-nNOS, Red-NeuN)

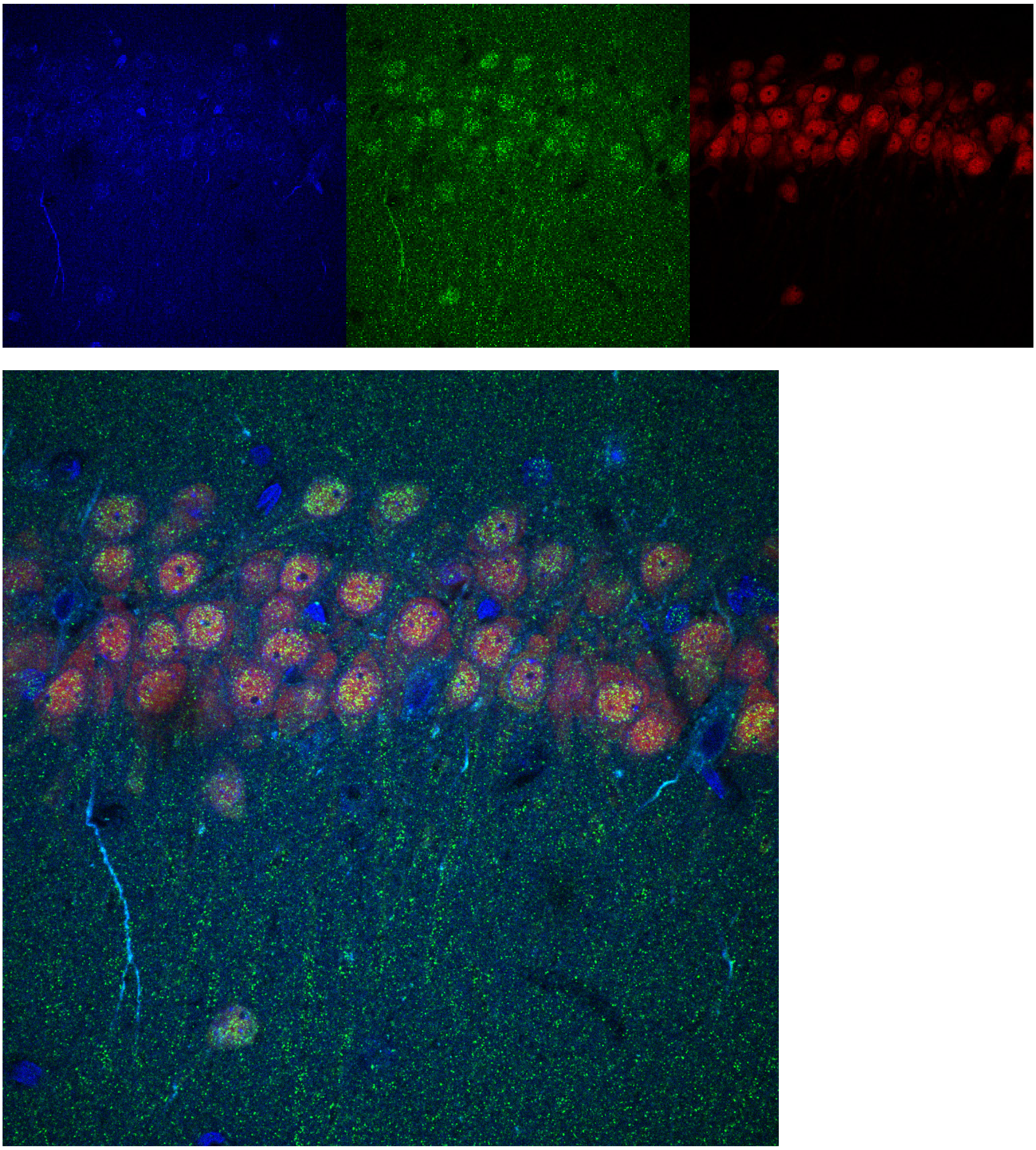

SHAM (DAPI-Blue, Green-nNOS, Red-NeuN)

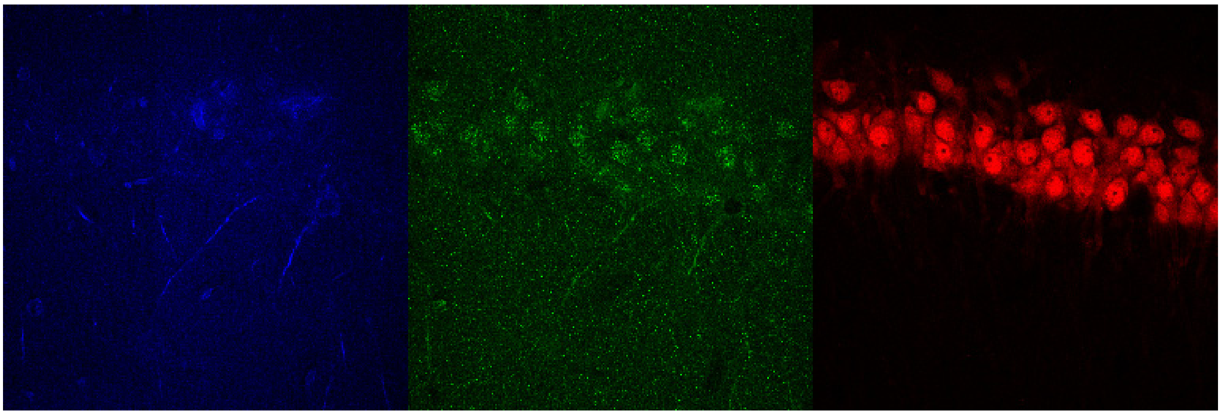

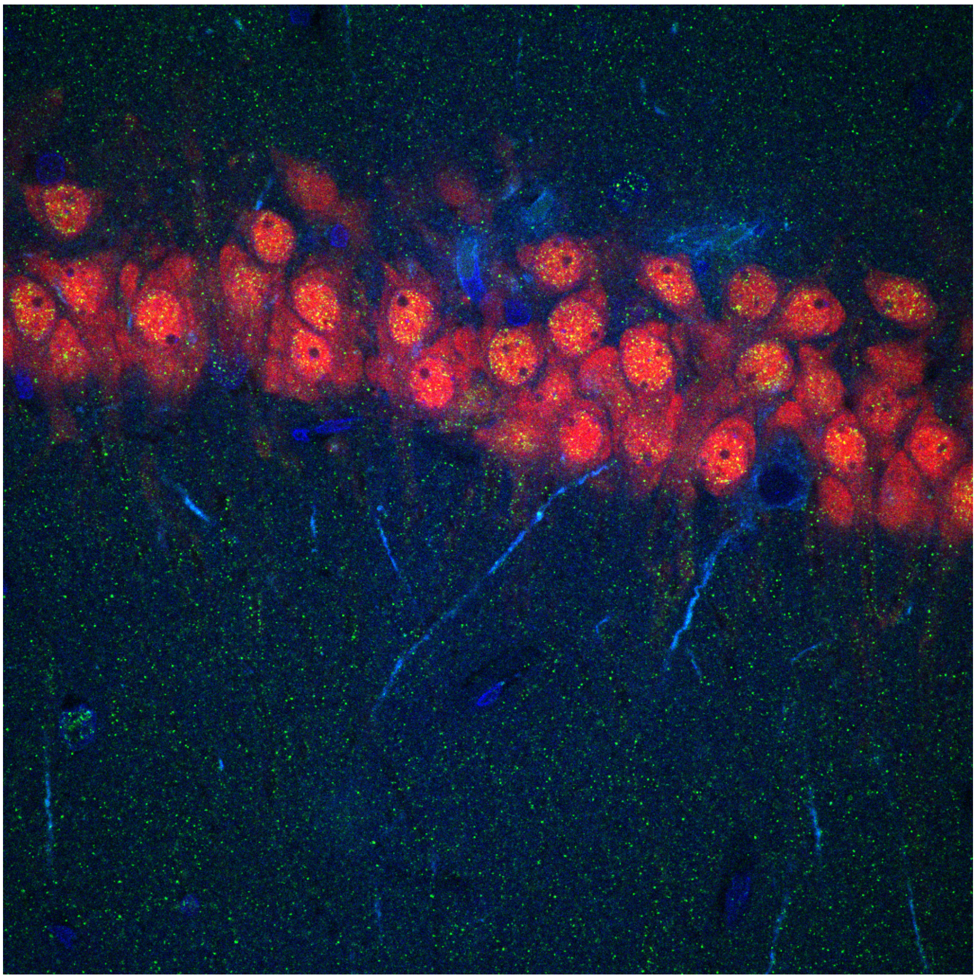

PVD-Perampanel (DAPI-Blue, Green-nNOS, Red-NeuN)

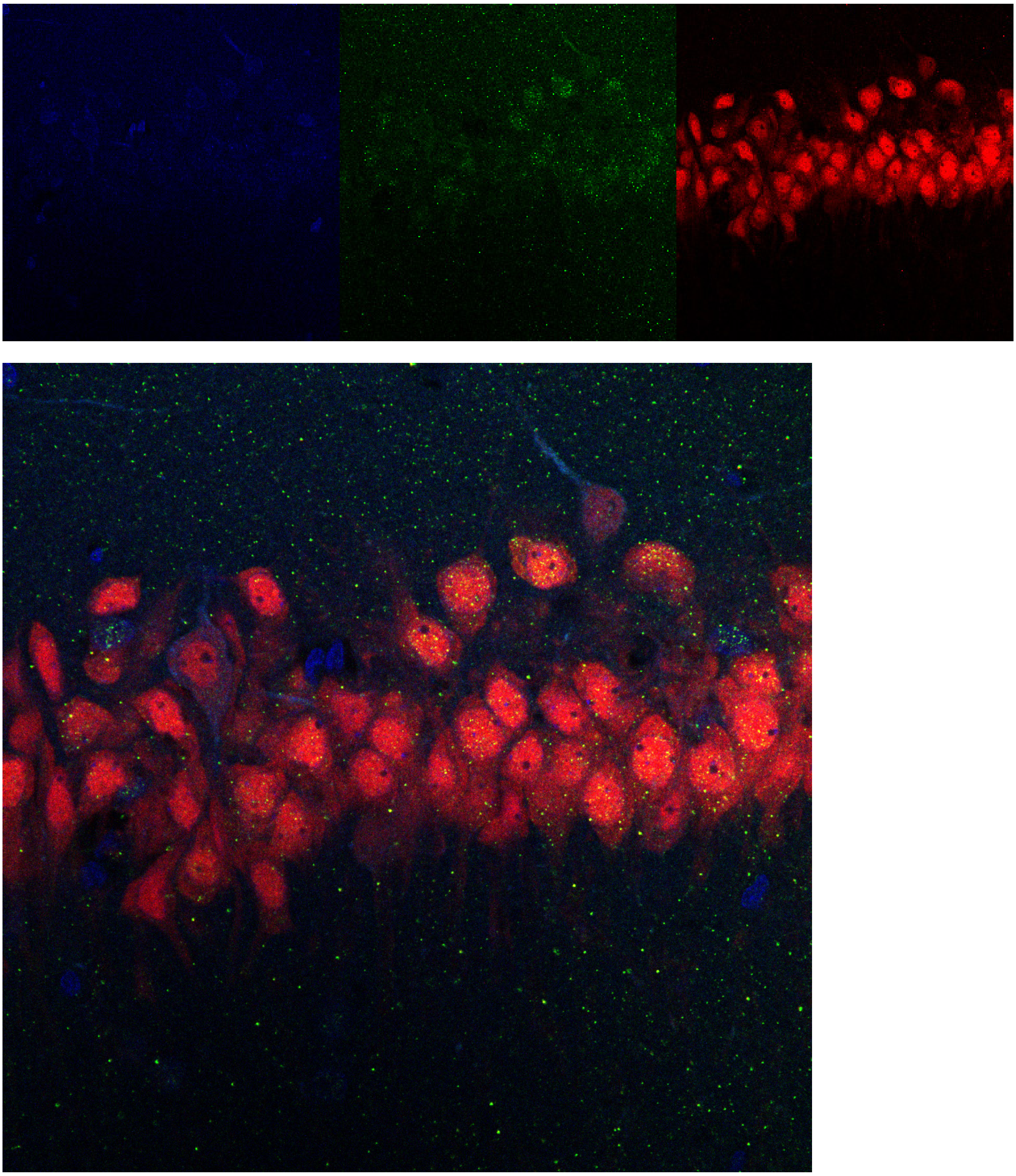

Supplement: Supplementary file 1 [file cells-14-01628-s001.zip › Supplementary Figure S3_NeuN and nNOS pilot.pdf]
